# *Sanjeevini: *a freely accessible web-server for target directed lead molecule discovery

**DOI:** 10.1186/1471-2105-13-S17-S7

**Published:** 2012-12-07

**Authors:** B Jayaram, Tanya Singh, Goutam Mukherjee, Abhinav Mathur, Shashank Shekhar, Vandana Shekhar

**Affiliations:** 1 Department of Chemistry, Indian Institute of Technology, Hauz Khas, New Delhi-110016, India; 2 Supercomputing Facility for Bioinformatics & Computational Biology, Indian Institute of Technology, Hauz Khas, New Delhi-110016, India; 3 Kusuma School of Biological Sciences, Indian Institute of Technology, Hauz Khas, New Delhi-110016, India

## Abstract

**Background:**

Computational methods utilizing the structural and functional information help to understand specific molecular recognition events between the target biomolecule and candidate hits and make it possible to design improved lead molecules for the target.

**Results:**

*Sanjeevini *represents a massive on-going scientific endeavor to provide to the user, a freely accessible state of the art software suite for protein and DNA targeted lead molecule discovery. It builds in several features, including automated detection of active sites, scanning against a million compound library for identifying hit molecules, all atom based docking and scoring and various other utilities to design molecules with desired affinity and specificity against biomolecular targets. Each of the modules is thoroughly validated on a large dataset of protein/DNA drug targets.

**Conclusions:**

The article presents *Sanjeevini*, a freely accessible user friendly web-server, to aid in drug discovery. It is implemented on a tera flop cluster and made accessible via a web-interface at http://www.scfbio-iitd.res.in/sanjeevini/sanjeevini.jsp. A brief description of various modules, their scientific basis, validation, and how to use the server to develop *in silico *suggestions of lead molecules is provided.

## Background

One of the main challenges in structure based drug discovery is to utilize the structural and chemical information of the drug targets and their ligand binding sites to create new molecules with high affinity and specificity, bioavailability and possibly least toxicity [1]. Computer aided drug discovery, in this context, is proving to be particularly invaluable [2-89]. The rapid ascent and acceptance of this methodology has been feasible due to advances in software and hardware. *Sanjeevini *server has been developed as an enabler for drug designers to address issues of affinity and selectivity of candidate molecules against drug targets with known structures. *Sanjeevini *comprises several modules with different functions, such as automated identification of potential binding sites (active sites) of ligands on the biomolecular target [90], a rapid screening of a million molecule database/natural product library [91] for identifying good candidates for any target protein, optimization of their geometries [92] and determination of partial atomic charges using quantum chemical methods [92,93], assignment of force field parameters to ligand [94] and the target protein/DNA [95], docking of the candidates in the active site of the drug target via *Monte Carlo *methods [90,96], estimation of binding free energies through empirical scoring functions [97-99], followed by rigorous analyses of the structure and energetics [100,101] of binding for further lead optimization. The computational pathway created rolls over into an automated pipe-line for lead design, if desired. The software takes three dimensional structure of the target protein or nucleotide sequence of DNA as an input; the remaining functionalities are built into the software suite to arrive at the structure and desired binding free energy of the protein/DNA-candidate molecule complex. The methodology treats biomolecular target and candidate molecules at the atomic level and solvent as a dielectric continuum. Validation studies on a large number of protein-ligand and DNA-ligand complexes suggest that performance of *Sanjeevini *is at the state of the art. The software is freely accessible over the net. We describe here as to how to harness the server for accelerating lead molecule discovery.

**Figure 1:**
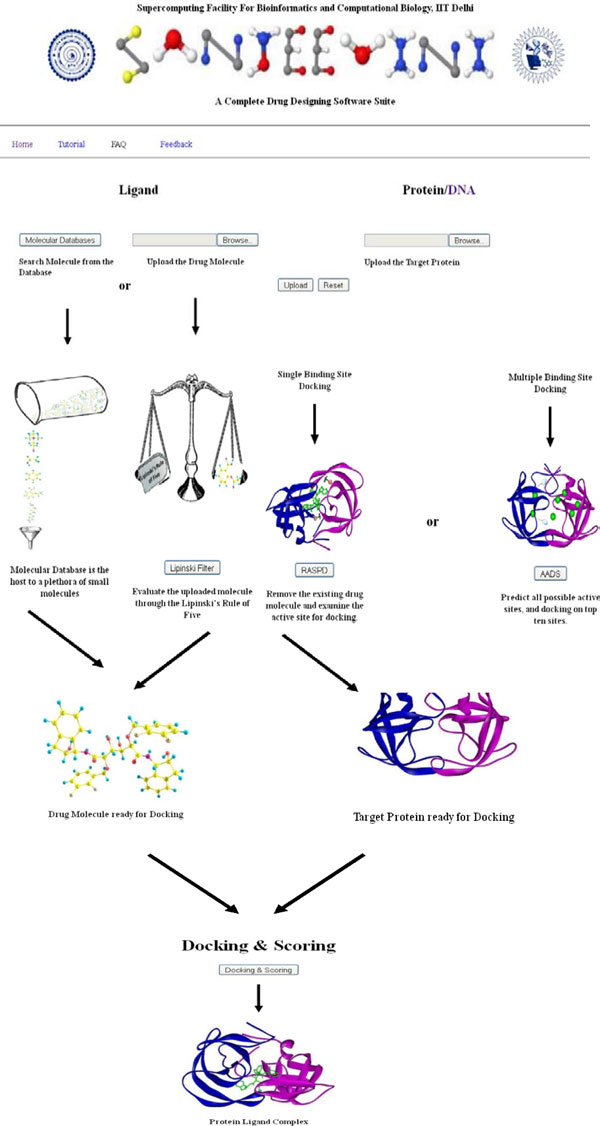
**A snapshot of the front-end of *Sanjeevini *web-server**.

**Figure 2:**
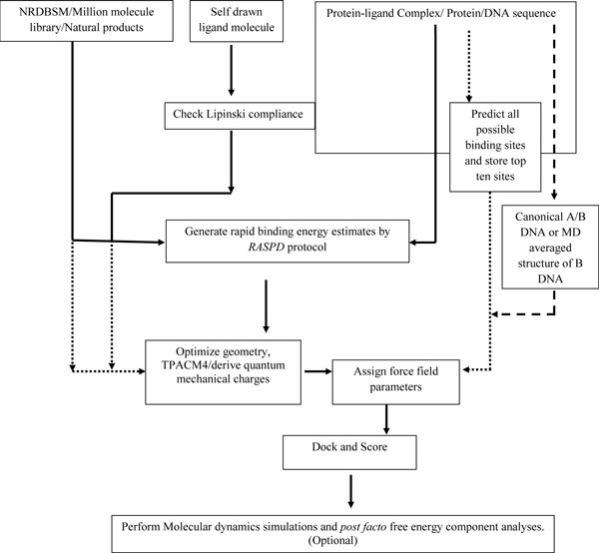
**Architecture of *Sanjeevini *Web-server**.

The front end of *Sanjeevini *website is shown in Figure 1 and the overall architecture of the software suite is given in Figure 2. *Sanjeevini *is a user friendly web interface where the demands on the user have been reduced to uploading of the target protein coordinates file or DNA sequence and the ligand molecule. The software protocol automatically standardizes the input formats of the biomolecule. Additionally, it determines the branch of pathway (Figure 2) that has to be followed (protein with known binding sites/protein with unknown binding site) by analyzing the target protein file and redirects the job instance for the same. Thus, any kind of overhead to the user to pre-format the input files for docking and scoring is removed. User can upload the desired ligand molecule either by drawing the molecule or by cultivating the molecular databases incorporated into *Sanjeevini*. There are three different molecular databases in-built in *Sanjeevini *namely *NRDBSM *containing 17000 molecules [82], a million molecule database containing one million small molecules, and a natural product database with 0.1 million natural products and their derivatives [91]. The molecules present in the database are Lipinski compliant [102,103]. *Sanjeevini *database of small organic molecules and the natural product database are localized on the linux clusters. Based on the user's choice of the physicochemical properties of interest including molecular weight, LogP, number of hydrogen bond donor and acceptor atoms, overall formal charge of the molecule and many more, a list of all the molecules falling in the ranges provided by the user are displayed in a downloadable form. However, if a self drawn molecule is uploaded by the user, then one can check its bioavailability by clicking the Lipinski's rule option in *Sanjeevini*. The program predicts the physico-chemical properties (Lipinski's rules) of the uploaded ligand molecule. If the binding site of the uploaded target protein is known and the coordinates of the protein-ligand complex are available in RCSB [104], then one can quickly check the binding affinity of the uploaded ligand and can also scan databases of small organic molecules [91] against any target protein by clicking the *RASPD *option (Mukherjee and Jayaram, Manuscript in preparation). The *RASPD *module takes 10-15 minutes in screening the database against a target protein. The docking and scoring module of *Sanjeevini *performs a series of computational steps such as preparation of the protein and the ligand from the files uploaded, docks the candidate molecule at the binding site via a *Monte Carlo *algorithm, minimizes and scores the docked complex, in an automated mode. The average time taken in the protein and ligand preparation and the *Monte Carlo *docking program ranges from 1-3 minutes. The *Monte Carlo *docking program is implemented in a parallel processing mode. The docked complexes are further minimized using the parallel version of Sander module of AMBER [105] which scales best on 32 processors. *Sanjeevini *programs run on linux clusters having infiniband network resources which facilitate a high through put distribution of the data across the various nodes. On an average, the total time taken by the complete docking and scoring protocol ranges from 5-20 minutes depending on the size of the protein and the ligand. The above time frames reported correspond to performance on a 32 processors cluster. A benchmark test on 8, 16 and 32 processors showed that the entire docking and scoring module scaled best on 32 processors. Memory consumption and I/O issues are minimal during program execution. The time taken also depends on the load on the server. Currently 80 processors are dedicated for jobs submitted to *Sanjeevini*. For each molecule five docked structures representing the poses of the molecule in the active site along with the binding affinity are emailed to user. However, if the binding sites are unknown in the protein, the *AADS *[90] option predicts ten hot spots/binding sites in the protein and docks the uploaded ligand molecule at all the ten predicted sites. Five docked structures representing the poses of the ligand molecule in the binding site along with their binding free energies are reported back to the user. The above docked structures may be treated as a reference protein-ligand complex which can be given as an input to scan the publicly accessible version of commercially-available compound database http://zinc.docking.org/ through *RASPD *protocol to arrive at suggestions of additional hit molecules against the target protein with unknown binding site information. A new cycle of design, docking and scoring for an iterative improvement of the candidate molecule can be initiated for desired affinities and scaffolds.

Target-molecule complexes with high binding affinity can be subjected to molecular dynamics simulations [101] in propitious cases, to investigate the effect of conformational flexibility, solvent, salt and entropic factors. About 100 or more structures may be collected over the trajectories and converged average binding free energies of the complexes may be obtained. Further *post facto *energy component analyses of the target-ligand complex can help in chemical modifications on the candidate molecule for enhancing the binding affinities. Different modules described above have been incorporated, which work in a pipeline as depicted in the architecture (Figure 2).

### A brief description of a few frequently used modules in Sanjeevini

*Sanjeevini *software comprises several modules with high accuracies, working in a pipeline, and given a protein/DNA as the drug target, and a ligand molecule which is optional to the software suite, it helps in designing lead molecules.

#### Scoring function

*Sanjeevini *comprises three scoring functions christened *Bappl *[97], *Bappl-Z *[98] and *PreDDICTA *[99] for protein-ligand complexes, Zn containing metalloproteinase-ligand complexes and DNA-ligand complexes respectively. *Bappl *is an all atom energy based empirical scoring function comprising electrostatics, van der Waals, desolvation and loss of conformational entropy of protein side chains upon ligand binding. *Bappl-Z *scores protein-ligand complexes with Zn as the metal ion in the binding site in which a non-bonded approach to model the interactions of the zinc ion with all other atoms of the protein-ligand complex has been employed along with the four terms described for *Bappl*. *PreDDICTA *is an all atom energy based scoring function which computes binding affinity of a DNA oligomer with a non-covalently bound drug molecule in the minor groove. The function is a combination of electrostatics, steric complementarities, entropic and solvent effects, including hydrophobicity. There are very few high accuracy scoring functions reported in literature for DNA-ligand complexes and, *PreDDICTA *thus provides a strong platform for designing molecules binding specifically to DNA. The program takes DNA-ligand complex as an input and outputs binding free energies associated with the complex.

#### Docking Module

The docking module of *Sanjeevini *comprises three programs christened *ParDOCK *[96], *AADS *[90] and *DNADock *[96,99]. *ParDock *is an all atom energy-based *Monte Carlo*, protein-ligand docking algorithm. The module requires a reference protein-ligand complex (target protein bound to a reference ligand at its binding site) as an input along with the candidate molecule to be docked. The algorithm docks the ligand molecule to the reference protein and outputs five docked structures representing different poses of ligand molecule along with the predicted binding free energies of the docked poses using *Bappl/BapplZ *scoring function. The program is in-built into *Sanjeevini *software for docking ligand molecules to the target protein for which crystal structure of the protein-ligand complex is available in literature. *AADS *(An automated active site identification, docking and scoring protocol for protein targets based on physico-chemical descriptors) predicts all potential binding sites in a protein and docks the input ligand molecule at the top ten predicted binding sites. Eight docked structures are generated at each of these ten sites and scored using *Bappl/BapplZ *scoring function. Five out of the eighty structures, favorable energetically are emailed back to the user along with the binding free energy values. The program has been tested previously [90] on more than 600 protein-ligand complexes with known binding site information. *AADS *predicted the true binding sites within the top ten sites with 100% accuracy. A blind docking on 170 protein targets [90] with known binding sites and known experimental binding free energies associated with the complexed ligands was also performed. The methodology restored the binding pose of the ligands to their native binding sites in the above 170 complexes with an accuracy of 90% for the top ranked docked structure and the predicted binding free energies of the top most docked structure correlated well with experiment (correlation coefficient ~ 0.82; see Figure F4 of [90]). The RMSD (Root Mean Square Deviation) between crystal and the docked structures in more than 80% of the cases is within 2 Å (Figure F5 of [90]). *DNADock *is an all atom *Monte Carlo *based docking algorithm which has been implemented in parallel mode and is incorporated into the software suite. The program takes nucleotide sequence and the candidate ligand molecule as input, generates canonical A or B DNA [123] or an average molecular dynamics B DNA structure [124,125] based on the user's choice, docks the candidate ligand molecule in the minor groove of DNA, and scores the docked structures through *PreDDICTA *scoring function. Five docked structures with their binding free energy values are reported back to the user.

*RASPD *(A rapid identification of hit molecules for target proteins via physico-chemical descriptors) is a computationally fast protocol for identifying hit molecules for any target protein. The methodology establishes complementarity in physico-chemical descriptor space of the target protein and the candidate molecule via a QSAR type approach and rapidly generates a reasonable estimate of the binding energy. The accuracies of *RASPD *are discussed elsewhere (Mukherjee and Jayaram manuscript in preparation).

## Results and discussion

**Figure 3:**
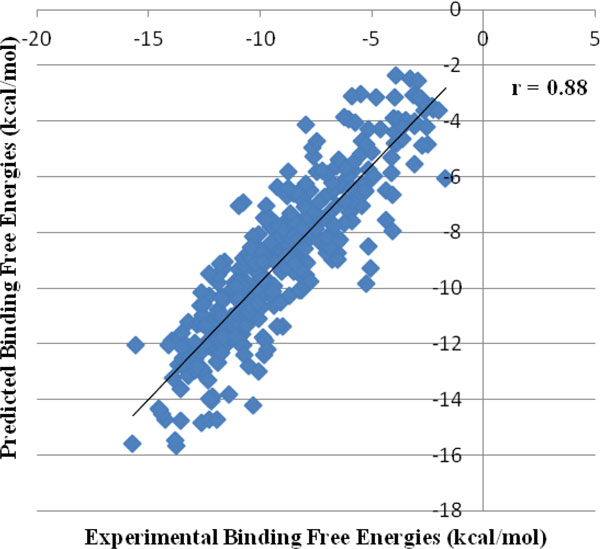
**Correlation between experimental and predicted binding free energies for 366 protein/DNA-ligand complexes**.

The scoring functions of *Sanjeevini *software were validated on a large dataset comprising 366 protein-ligand complexes, Zn-containing metalloproteinase-ligand complexes and DNA-ligand complexes which includes 335 crystal structures and 31 modeled structures. The PDB IDs of the validation dataset with the experimental and predicted binding free energies are provided in Additional file 1. A correlation coefficient of r = 0.88 was obtained between the experimental and predicted binding free energies on the above dataset as shown in Figure 3.

Some of the published results of scoring functions for protein-ligand complexes originating in physics based or knowledge based methods include DFIRE (r = 0.63) [106], × SCORE (r = 0.77) [107], SMoG (r = 0.79) [108], BLEEP (r = 0.74) [109], PMF(r = 0.78) [110], SCORE (r = 0.81) [111], LUDI (r = 0.83) [112], ChemScore (r = 0.84) [113], Ligscore (r = 0.87) [114], KGS comprising of both X-Score and PLP (r = 0.82) [115]. *Sanjeevini *scoring function for protein-ligand complexes yielded a correlation coefficient (r) of 0.87. There are very few scoring functions reported in literature for DNA-ligand complexes. One among them is the KS score (r = 0.68) [116]. *Sanjeevini *scoring function for DNA-ligand complexes has been tested on 39 DNA-ligand complexes involving no training which yielded a correlation coefficient of 0.90. *PreDDICTA *has been reported to perform better than some of the existing scoring functions for DNA-ligand complexes in literature [116]. Scoring functions for zinc containing metalloprotein-ligand complexes reported in literature include the work of Raha et al., (R^2 ^= 0.69) [117], Hou et al., (R^2 ^= 0.85) [118], Hu et al., (0.50) [119], Rizzo et al., (R^2 ^= 0.74) [120], Khandelwal et al., (R^2 ^= 0.90) [121]. *Sanjeevini *yielded a correlation coefficient R^2 ^= 0.82 on zinc-containing metalloprotein ligand complexes. The overall correlation coefficient of *Sanjeevini *for protein/DNA-ligand complexes (Figure 3) is 0.88.

**Figure 4:**
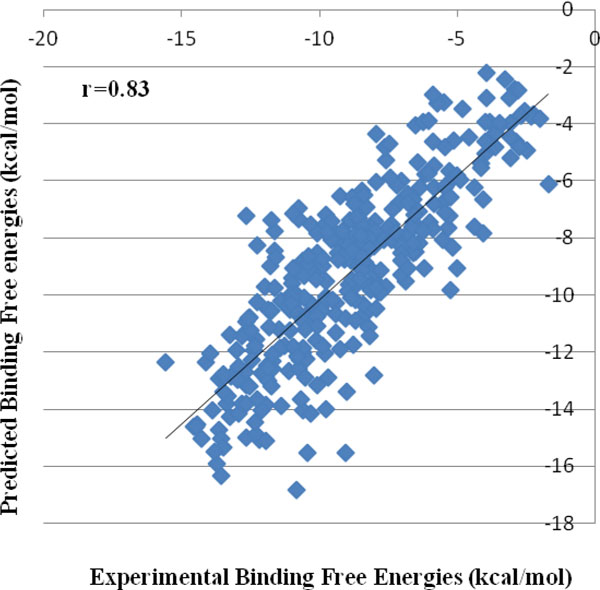
**Correlation between experimental and predicted binding free energies of the top ranked docked structures for 335 protein/DNA-ligand complexes**.

**Figure 5:**
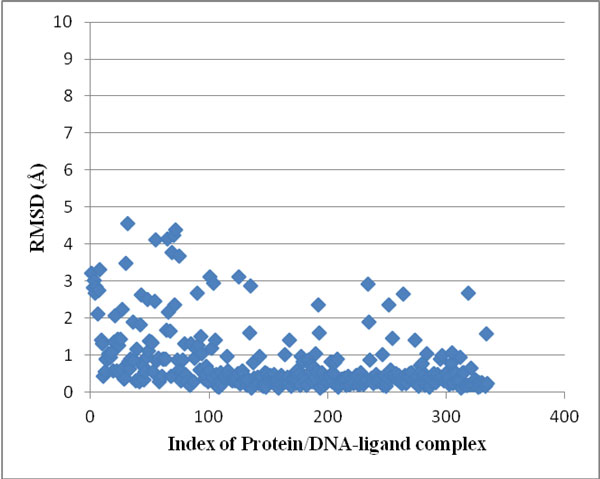
**Root Mean Square Deviation between the crystal structure and the top ranked docked structure for 335 protein/DNA-ligand complexes**.

The docking module of *Sanjeevini *has been validated on a dataset of 335 DNA/protein targets with known binders and structures and known experimental binding free energies. The predicted binding free energies of the top ranked docked structures reported by *Sanjeevini *(Additional File 2) were compared with experiment (Figure 4) and also the RMSDs (root mean square deviations) between the crystal structures and the top ranked docked structures (Figure 5). The high accuracies obtained by *Sanjeevini *as evident from a correlation coefficient of r = 0.83 in Figure 4 and RMSDs lying within 2 Å in Figure 5, provide a strong platform to design drug-like molecules. For protein-ligand complexes Autodock Vina [5] has been reported to predict the top most structure within 2Å RMSD from the native complex with 80% accuracy. In a recent work of Zhong-Ru Xie et al. DrugScore^CSD ^scoring function was compared with some of the known scoring functions in literature [122] and was reported to perform better than others giving an accuracy of 87% in predicting the top most docked structure within an RMSD of 2Å from crystal structure. The docking and the scoring module of *Sanjeevini *yielded 90% accuracy in predicting the top most docked structure within 2Å RMSD from crystal structure on a large dataset (335 complexes: Figure 5).

### Case studies

While designing new molecules for a target protein/DNA, user may have experimental (K_i_/IC50/K_d_) values of known binders reported in the literature. Before designing new candidate molecules against a target protein/DNA, we propose to the *Sanjeevini *user to predict the binding free energies of the known binders and plot a correlation graph between the experimental and predicted binding free energies. This would give a relative understanding of the predicted binding free energies vis-a-vis experiment, helping in discriminating between drug-like and non-drug-like molecules against a given target. With this proposal, we present a few case studies on an important class of drug targets which can set examples for the *Sanjeevini *users to utilize the same methodology on various drug targets to come up with suggestions of hit molecules.

#### Case 1: Protein targets with known binding site information

**Figure 6:**
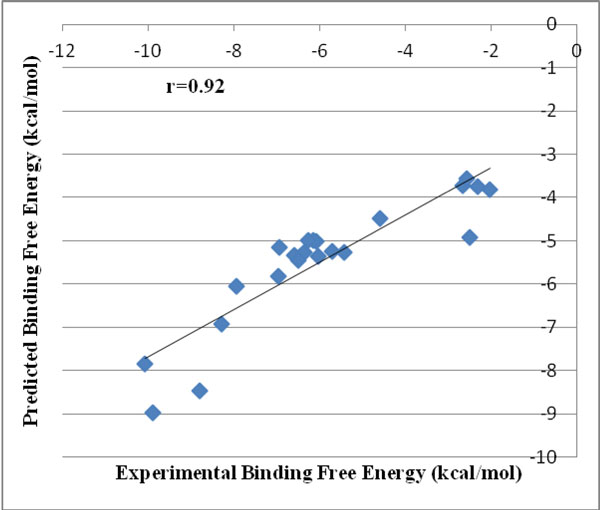
**Correlation between experimental and predicted binding free energies of the top ranked docked structures for 23 trypsin binding molecules with known binding site information in the target protein**.

Majority of drugs deposited in RCSB have been co-crystallized with a single protein or more than one protein [126] yielding the drug binding site for the target protein. The first case study was on protein targets for which structures of the protein-ligand complexes were available in the database specifying the binding site. Serine proteinases play an important role in many biological processes [127]. For instance trypsin helps in digestion and thrombins in the blood coagulation cascade. The above class of enzymes is implicated in a wide spectrum of diseases which are related to a malfunctioning in this regulation. We predicted the binding energies of 12 trypsin binding molecules. In addition, some of the known synthetic inhibitors [128] of bovine pancreatic trypsins, PDBID 1S0R were also docked and scored. The predicted binding free energies associated with the top ranked docked complex for all the above data are shown in Table 1. A correlation coefficient of r = 0.92 was obtained between the experimental and predicted binding free energies as illustrated in Figure 6.

**Table 1:** Docking and scoring studies of experimentally reported trypsin binding molecules using *Sanjeevini*

**Sl. No**.	PDBID^a^	Ligand (Molecular formula)	EXBFE (kcal/mol)#	PBFE (kcal/mol)^b^	PBFE (kcal/mol)^c^
1	1BRA	C_7_H_8_N_2_	-2.496	-4.92	-4.59
2	1F0T	C_19_H_19_N_5_O_4_S_2_	-8.29	-6.92	-7.35
3	1F0U	C_27_H_30_N_4_O_3_	-9.89	-8.98	-6.96
4	1MTW	C_26_H_28_N_4_O_3_	-10.076	-7.84	-6.64
5	1PPC	C_27_H_31_N_5_O_4_S	-8.8	-8.46	-6.85
6	1TNH	C_7_H_9_FN	-4.59	-4.49	-4.28
7	1TNI	C_10_H_15_N	-2.32	-3.74	-3.53
8	1TNJ	C_8_H_12_N	-2.67	-3.72	-3.77
9	1TNK	C_9_H_14_N	-2.03	-3.81	-3.51
10	1TNL	C_9_H_12_N	-2.56	-3.56	-3.99
11	1TPP	C_10_H_12_N_2_O_3_	-7.95	-6.05	-5.55
12	3PTB	C_7_H_8_N_2_	-6.46	-5.36	-5.27
13	1S0R/1S0Q	C_8_H_10_N_3_O_1_	-5.71	-5.25	-5.59
14	1S0R/1S0Q	C8H11N2O1	-6.04	-5.37	-5.51
15	1S0R/1S0Q	C7H10N3	-6.95	-5.16	-5.99
16	1S0R/1S0Q	C7H9N2	-6.35	-5.26	-5.12
17	1S0R/1S0Q	C8H11N2	-6.59	-5.33	-5.56
18	1S0R/1S0Q	C9H13N2	-6.07	-5.01	-5.41
19	1S0R/1S0Q	C10H15N2	-6.14	-5	-5.55
20	1S0R/1S0Q	C_10_H_15_N_2 _(iso)	-5.42	-5.26	-5.79
21	1S0R/1S0Q	C_11_H_17_N_2_	-6.26	-5	-6.08
22	1S0R/1S0Q	C_12_H_19_N_2_	-6.5	-5.45	-6.55
23	1S0R/1S0Q	C_13_H_21_N_2_	-6.97	-5.82	-6.53

# Experimental binding free energies of trypsin binding molecules.

^a ^Protein Data Bank ID

^b ^Predicted binding free energies of trypsin binding molecules in Case Study 1.

^c ^Predicted binding free energies of trypsin binding molecules in Case Study 2.

#### Case 2: Input as a target protein with unknown binding site and a candidate ligand

**Figure 7:**
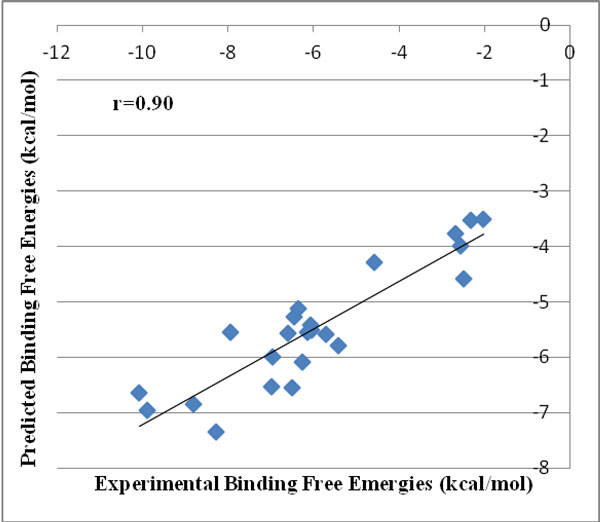
**Correlation between experimental and predicted binding free energies of the top ranked docked structures for 23 trypsin binding molecules with unknown binding site information in the target protein**.

When the user has the 3D coordinates of a target protein, either as deposited in the protein data bank or as a modeled structure with no binding site information, the *AADS *pathway of *Sanjeevini *gets pre-selected to come up with suggestions of hit molecules. We performed a case study on the trypsin binding inhibitors considered in the first case study. For the twelve protein structures complexed with ligand and known binding site information, we deliberately removed the ligands from the target proteins and uploaded the target to *Sanjeevini *for a blind docking with the ligand. For Bovine pancreatic trypsin receptor, a structure with unknown binding site information (PDBID 1S0Q) is also available in the literature [128,129] along with a protein-ligand complex (PDBID 1S0R) which was taken as an input in the first case study. The target receptor with unknown binding site and its synthetic inhibitors were given as input to *Sanjeevini*. *AADS *module gave an output of five docked structures along with binding free energies. A total of 230 docking runs corresponding to 10 binding sites for each target were performed in an automated mode by *Sanjeevini *in the above case study for the 23 trypsin binding molecules. We compared the predicted binding free energies of the energetically top ranked structure for each target (shown in Table 1) and plotted a correlation graph between the experimental and predicted binding free energies (shown in Figure 7).

In the Bovine pancreatic trypsins, the amino acids mainly involved in interactions with the ligand molecules are reported to be Ser 172, Asp 171 and Gly 196 in the target protein (PDBID 1S0R) [104]. We visualized the docked structures obtained from the above blind docking studies of trypsin inhibitors against the target (PDB ID 1S0Q) to make sure if the top ranked docked structures have the native ligand pose restored in the native binding site of target. A good estimate of the binding free energies through *Sanjeevini *protocol in the above two case studies evident from a high correlation coefficient obtained (Figures 6 and 7) by two different methodologies taking care of inputs with known binding site and unknown binding site information in a protein target illustrates the strength of the *Sanjeevini *software.

### Future directions of *Sanjeevini*

Improvements conceived in the future versions of *Sanjeevini *are: (i) consideration of the flexibility of the candidate ligand molecules, and the active site amino acids of the target, (ii) docking and scoring of the candidate molecules in the presence of a cofactor or multiple metal ions, (iii) extension of the DNA docking and scoring methodology to DNA binding intercalators and eventually (iv) creating an assembly line from genomes to hits [130].

## Conclusions

This article presents *Sanjeevini*, a state of the art, structure based computer aided drug discovery (SBDD/CADD) software suite implemented on an 80 processor cluster and presented to the user as a freely accessible server. The high accuracy of the modules and a user friendly environment should help the user in designing novel lead compounds.

## Availability and requirements

Project name: *Sanjeevini*

Project home page: http://www.scfbio-iitd.res.in/sanjeevini/sanjeevini.jsp

Operating systems: Linux

Programming languages: C++ and java

Any restrictions to use by non-academics: none

A detailed tutorial with various inputs and outputs of *Sanjeevini *in the form of snapshots is available at the following link http://www.scfbio-iitd.res.in/sanjeevini/example/Tutorial.pdf. The coordinates of the validation dataset of 335 protein/DNA targets are available at the following link http://www.scfbio-iitd.res.in/sanjeevini/dataset.jsp.

## Competing interests

The authors declare that they have no competing interests.

## Authors' contributions

BJ designed the study. TS, GM and AM collected the data. BJ & TS analyzed the data and wrote the manuscript. SS & VS web-enabled the software.

## Supplementary Material

Additional file 1 **Validation of *Sanjeevini *scoring function on 366 Protein/DNA-ligand complexes**. Click here for file

Additional file 2 Docking and scoring studies on 335 Protein/DNA drug targets via *Sanjeevini*. Click here for file

## References

[B1] Shaikh S Jain T Sandhu G Latha N Jayaram B A physico-chemical pathway from targets to leads Current Pharmaceutical Design 2007 13 3454 3470 10.2174/138161207782794220 18220783

[B2] Yamada M Itai A Development of an efficient automated docking method Chem Pharm Bull 1993 41 1200 1202 10.1248/cpb.41.1200

[B3] Mizutani MY Tomioka N Itai A Rational automatic search method for stable docking models of protein and ligand J Mol Biol 1994 243 310 326 10.1006/jmbi.1994.1656 7932757

[B4] Mizutani MY Takamatsu Y Ichinose T Nakamura K Itai A Effective handling of induced-fit motion in flexible docking Proteins Struct Funct Genet 2006 63 878 891 10.1002/prot.20931 16532451

[B5] Morris GM Goodsell DS Halliday RS Huey R Hart WE Belew RK Olson AJ Automated docking using a Lamarckian genetic algorithm and an empirical binding free energy function J Comput Chem 1998 19 1639 1662 10.1002/(SICI)1096-987X(19981115)19:14<1639::AID-JCC10>3.0.CO;2-B

[B6] Osterberg F Morris GM Sanner MF Olson AJ Goodsell DS Automated docking to multiple target structures: incorporation of protein mobility and structural water heterogeneity in autodock Proteins Struct Funct Genet 2002 46 34 40 10.1002/prot.10028 11746701

[B7] Trott O Olson AJ AutoDock Vina: improving the speed and accuracy of docking with a new scoring function, efficient optimization and multithreading Journal of Computational Chemistry 2010 31 455 461 19499576 10.1002/jcc.21334 PMC3041641

[B8] Wu G Robertson DH Brooks CL III Vieth M Detailed analysis of grid-based molecular docking: a case study of CDOCKER--a CHARMm-based MD docking algorithm J Comput Chem 2003 24 1549 1562 10.1002/jcc.10306 12925999

[B9] Vieth M Hirst JD Kolinski A Brooks CL III Assessing energy functions for flexible docking J Comput Chem 1998 19 1612 1622 10.1002/(SICI)1096-987X(19981115)19:14<1612::AID-JCC7>3.0.CO;2-M

[B10] Lawrence MC Davis PC CLIX: a search algorithm for finding novel ligands capable of binding proteins of known three-dimensional structure Proteins Struct Funct Genet 1992 12 31 41 10.1002/prot.340120105 1313175

[B11] Taylor JS Burnett RM DARWIN: a program for docking flexible molecules Proteins Struct Funct Genet 2000 41 173 191 10.1002/1097-0134(20001101)41:2<173::AID-PROT30>3.0.CO;2-3 10966571

[B12] Clark KP Jain AN Flexible ligand docking without parameter adjustment across four ligand-receptor complexes J Comput Chem 1995 16 1210 1226 10.1002/jcc.540161004

[B13] Rohs R Bloch I Sklenar H Shakked Z Molecular flexibility in ab-initio drug docking to DNA: Binding-site and binding-mode transitions in all-atom Monte Carlo simulations Nucleic Acids Res 1995 33 7048 7057 16352865 10.1093/nar/gki1008 PMC1312361

[B14] Oshiro CM Kuntz ID Dixon JS Flexible ligand docking using a genetic algorithm J Comput Aided Mol Des 1995 9 113 130 10.1007/BF00124402 7608743

[B15] Knegtel RMA Kuntz ID Oshiro CM Molecular docking to ensembles of protein structures J Mol Biol 1997 266 424 440 10.1006/jmbi.1996.0776 9047373

[B16] Kang X Shafer RH Kuntz ID Calculation of ligand-nucleic acid binding free energies with the generalized-born model in DOCK Biopolymers 2004 73 192 204 10.1002/bip.10541 14755577

[B17] Moustakas DT Lang PT Pegg S Pettersen E Kuntz ID Brooijmans N Rizzo RC Development and validation of a modular, extensible docking program: DOCK 5 J Comput Aided Mol Des 2006 20 601 619 10.1007/s10822-006-9060-4 17149653

[B18] Irwin JJ Shoichet BK Mysinger MM Huang N Colizzi F Wassam P Cao Y Automated docking screens: a feasibility study J Med Chem 2009 52 5712 5720 10.1021/jm9006966 19719084 PMC2745826

[B19] Hart TN Read RJ A multiple-start Monte Carlo docking method Proteins Struct Funct Genet 1992 13 206 222 10.1002/prot.340130304 1603810

[B20] Vieth M Cummins DJ DoMCoSAR: a novel approach for establishing the docking mode that is consistent with the structure--activity relationship. Application to HIV-1 protease inhibitors and VEGF receptor tyrosine kinase inhibitors J Med Chem 2000 43 3020 3032 10.1021/jm990609e 10956210

[B21] Schafferhans A Klebe G Docking ligands onto binding site representations derived from proteins built by homology modelling J Mol Biol 2001 307 407 427 10.1006/jmbi.2000.4453 11243828

[B22] Grosdidier A Zoete V Michielin O EADock: docking of small molecules into protein active sites with a multiobjective evolutionary optimization Proteins Struct Funct Genet 2007 67 1010 1025 10.1002/prot.21367 17380512

[B23] Zsoldos Z Reid D Simon A Sadjad BS Johnson AP eHiTS: an innovative approach to the docking and scoring function problems Curr Protein Pept Sci 2006 7 421 435 10.2174/138920306778559412 17073694

[B24] Pang YP Perola E Xu R Prendergast FG EUDOC: a computer program for identification of drug interaction sites in macromolecules and drug leads from chemical databases J Comput Chem 2001 22 1750 1771 10.1002/jcc.1129 12116409

[B25] Taylor RD Jewsbury PJ Essex JW FDS: flexible ligand and receptor docking with a continuum solvent model and soft-core energy function J Comput Chem 2003 24 1637 1656 10.1002/jcc.10295 12926007

[B26] Majeux N Scarsi M Apostolakis J Ehrhardt C Caflisch A Exhaustive docking of molecular fragments with electrostatic solvation Proteins Struct Funct Genet 1999 37 88 105 10451553

[B27] Budin N Majeux N Caflisch A Fragment-based flexible ligand docking by evolutionary optimization Biol Chem 2001 382 1365 1372 11688719 10.1515/BC.2001.168

[B28] Kolb P Caflisch A Automatic and efficient decomposition of two-dimensional structures of small molecules for fragment-based high-throughput docking J Med Chem 2006 49 7384 7392 10.1021/jm060838i 17149868

[B29] Corbeil CR Englebienne P Moitessier N Docking ligands into flexible and solvated macromolecules. 1. Development and validation of FITTED 1.0 J Chem Inf Model 2007 47 435 449 10.1021/ci6002637 17305329

[B30] Rarey M Kramer B Lengauer T Klebe GA Fast flexible docking method using an incremental construction algorithm J Mol Biol 1996 261 470 489 10.1006/jmbi.1996.0477 8780787

[B31] Rarey M Kramer B Lengauer T The particle concept: placing discrete water molecules during protein-ligand docking predictions Proteins Struct Funct Genet 1999 34 17 28 10.1002/(SICI)1097-0134(19990101)34:1<17::AID-PROT3>3.0.CO;2-1 10336380

[B32] Clausen H Buning C Rarey M Lengauer T FLEXE: efficient molecular docking considering protein structure variations J Mol Biol 2001 308 377 395 10.1006/jmbi.2001.4551 11327774

[B33] Zhao Y Sanner MF FLIPDock: docking flexible ligands into flexible receptors Proteins Struct Funct Bioinf 2007 68 726 737 10.1002/prot.21423 17523154

[B34] Miller MD Kearsley SK Underwood DJ Sheridan RP FLOG: a system to select 'quasi-flexible' ligands complementary to a receptor of known three-dimensional structure J Comput Aided Mol Des 1994 8 153 174 10.1007/BF00119865 8064332

[B35] McGann MR Almond HR Nicholls A Grant JA Brown FK Gaussian docking functions Biopolymers 2003 68 76 90 10.1002/bip.10207 12579581

[B36] Gabb HA Jackson RM Sternberg MJE Modelling protein docking using shape complementarity, electrostatics and biochemical information J Mol Biol 1997 272 106 120 10.1006/jmbi.1997.1203 9299341

[B37] Charifson PS Corkery JJ Murcko MA Walters WP Consensus scoring: a method for obtaining improved hit rates from docking databases of three-dimensional structures into proteins J Med Chem 1999 42 5100 5109 10.1021/jm990352k 10602695

[B38] Li H Li C Gui C Luo X Chen K Shen J Wang X Jiang H GAsDock: a new approach for rapid flexible docking based on an improved multi-population genetic algorithm Bioorg Med Chem Lett 2004 14 4671 4676 10.1016/j.bmcl.2004.06.091 15324886

[B39] Yang JM Chen CC GEMDOCK: a generic evolutionary method for molecular docking Proteins Struct Funct Bioinf 2004 55 288 304 10.1002/prot.20035 15048822

[B40] Tietze S Apostolakis J GlamDock: development and validation of a new docking tool on several thousand protein-ligand complexes J Chem Inf Model 2007 47 1657 1672 10.1021/ci7001236 17585857

[B41] Friesner RA Banks JL Murphy RB Halgren TA Klicic JJ Mainz DT Repasky MP Knoll EH Shelley M Perry JK Shaw DE Francis P Shenkin PS Glide: a new approach for rapid, accurate docking and scoring. 1. Method and assessment of docking accuracy J Med Chem 2004 47 1739 1749 10.1021/jm0306430 15027865

[B42] Sherman W Day T Jacobson MP Friesner RA Farid R Novel procedure for modeling ligand/receptor induced fit effects J Med Chem 2006 49 534 553 10.1021/jm050540c 16420040

[B43] Verdonk ML Cole JC Hartshorn MJ Murray CW Taylor RD Improved protein-ligand docking using GOLD Proteins Struct Funct Genet 2003 52 609 623 10.1002/prot.10465 12910460

[B44] Verdonk ML Chessari G Cole JC Hartshorn MJ Murray CW Nissink JWM Taylor RD Taylor R Modeling water molecules in protein-ligand docking using GOLD J Med Chem 2005 48 6504 6515 10.1021/jm050543p 16190776

[B45] Welch W Ruppert J Jain AN Hammerhead: fast, fully automated docking of flexible ligands to protein binding sites Chem Biol 1996 3 449 462 10.1016/S1074-5521(96)90093-9 8807875

[B46] Dominguez C Boelens R Bonvin AMJJ HADDOCK: a protein-protein docking approach based on biochemical or biophysical information J Am Chem Soc 2003 125 1731 1737 10.1021/ja026939x 12580598

[B47] Floriano WB Vaidehi N Zamanakos G Goddard WA III HierVLS hierarchical docking protocol for virtual ligand screening of large-molecule databases J Med Chem 2004 47 56 71 10.1021/jm030271v 14695820

[B48] Trabanino RJ Hall SE Vaidehi N Floriano WB Kam VWT Goddard WA III First principles predictions of the structure and function of G-protein-coupled receptors: validation for bovine rhodopsin Biophys J 2004 86 1904 1921 10.1016/S0006-3495(04)74256-3 15041637 PMC1304048

[B49] Abagyan R Totrov M Kuznetsov D ICM--a new method for protein modeling and design: applications to docking and structure prediction from the distorted native conformation J Comput Chem 1994 15 488 506 10.1002/jcc.540150503

[B50] Totrov M Abagyan R Flexible protein-ligand docking by global energy optimization in internal coordinates Proteins Struct Funct Genet 1997 29 215 220 10.1002/(SICI)1097-0134(1997)1+<215::AID-PROT29>3.0.CO;2-Q 9485515

[B51] Diller DJ Merz KM Jr High throughput docking for library design and library prioritization Proteins Struct Funct Genet 2001 43 113 124 10.1002/1097-0134(20010501)43:2<113::AID-PROT1023>3.0.CO;2-T 11276081

[B52] Wu SY McNae I Kontopidis G McClue SJ McInnes C Stewart KJ Wang S Zheleva DI Marriage H Lane DP Taylor P Fischer PM Walkinshaw MD Discovery of a novel family of CDK inhibitors with the program LIDAEUS: structural basis for ligand-induced disordering of the activation loop Structure 2003 11 399 410 10.1016/S0969-2126(03)00060-1 12679018

[B53] Sobolev V Wade RC Vriend G Edelman M Molecular docking using surface complementarity Proteins Struct Funct Genet 1996 25 120 129 10.1002/(SICI)1097-0134(199605)25:1<120::AID-PROT10>3.3.CO;2-1 8727324

[B54] Fradera X Kaur J Mestres J Unsupervised guided docking of covalently bound ligands J Comput Aided Mol Des 2004 18 635 650 10.1007/s10822-004-5291-4 15849994

[B55] Liu M Wang S MCDOCK: a Monte Carlo simulation approach to the molecular docking problem J Comput Aided Mol Des 1999 13 435 451 10.1023/A:1008005918983 10483527

[B56] Thomsen R Christensen MH MolDock: A new technique for high-accuracy molecular docking J Med Chem 2006 49 3315 3321 10.1021/jm051197e 16722650

[B57] Schneidman-Duhovny D Inbar Y Nussinov R Wolfson HJ PatchDock and SymmDock: servers for rigid and symmetric docking Nucleic Acids Res 2005 33 363 367 10.1093/nar/gki481 PMC1160241 15980490

[B58] Tøndel K Anderssen E Drabløs F Protein Alpha Shape (PAS) Dock: A new gaussian-based score function suitable for docking in homology modelled protein structures J Comput Aided Mol Des 2006 20 131 144 10.1007/s10822-006-9041-7 16652207

[B59] Joseph-McCarthy D Thomas BE IV Belmarsh M Moustakas D Alvarez JC Pharmacophore-based molecular docking to account for ligand flexibility Proteins Struct Funct Gene 2003 51 172 188 10.1002/prot.10266 12660987

[B60] Goto J Kataoka R Hirayama N Ph4Dock: pharmacophorebased protein-ligand docking J Med Chem 2004 4 6804 6811 15615529 10.1021/jm0493818

[B61] Kozakov D Brenke R Comeau SR Vajda S PIPER: an FFTbased protein docking program with pairwise potentials Proteins Struct Funct Genet 2006 65 392 406 10.1002/prot.21117 16933295

[B62] Korb O Stutzle T Exner TE PLANTS: application of ant colony optimization to structure-based drug design Lecture Notes in Computer Science (Including Subseries Lecture Notes in Artificial Intelligence and Lecture Notes in Bioinformatics). Brussels 2006 247 258

[B63] Trosset JY Scheraga HA PRODOCK: software package for protein modeling and docking J Comput Chem 1999 20 412 427 10.1002/(SICI)1096-987X(199903)20:4<412::AID-JCC3>3.0.CO;2-N

[B64] Murray CW Baxter CA Frenkel AD The sensitivity of the results of molecular docking to induced fit effects: application to thrombin, thermolysin and neuraminidase J Comput Aided Mol Des 1999 13 547 562 10.1023/A:1008015827877 10584214

[B65] Seifert MHJ ProPose: steered virtual screening by simultaneous protein-ligand docking and ligand-ligand alignment J Chem Inf Model 2005 45 449 460 10.1021/ci0496393 15807511

[B66] Pei J Wang Q Liu Z Li Q Yang K Lai L PSI-DOCK: towards highly efficient and accurate flexible ligand docking Proteins Struct Funct Genet 2006 62 934 946 10.1002/prot.20790 16395666

[B67] Jackson RM Q-fit: a probabilistic method for docking molecular fragments by sampling low energy conformational space J Comput Aided Mol Des 2002 16 43 57 10.1023/A:1016307520660 12197665

[B68] McMartin C Bohacek RS QXP: powerful, rapid computer algorithms for structure-based drug design J Comput Aided Mol Des 1997 11 333 344 10.1023/A:1007907728892 9334900

[B69] Morley SD Afshar M Validation of an empirical RNA-ligand scoring function for fast flexible docking using RiboDocks J Comput Aided Mol Des 2004 18 189 208 15368919 10.1023/b:jcam.0000035199.48747.1e

[B70] Meiler J Baker D ROSETTALIGAND: protein-small molecule docking with full side-chain flexibility Proteins Struct Funct Genet 2006 65 538 548 10.1002/prot.21086 16972285

[B71] Burkhard P Taylor P Walkinshaw MD An example of a protein ligand found by database mining: description of the docking method and its verification by a 2.3A° X-ray structure of a thrombin-ligand complex J Mol Biol 1998 277 449 466 10.1006/jmbi.1997.1608 9514757

[B72] Wu G Vieth M SDOCKER: a method utilizing existing X-ray structures to improve docking accuracy J Med Chem 2004 47 3142 3148 10.1021/jm040015y 15163194

[B73] Schnecke V Kuhn LA Virtual screening with solvation and ligand-induced complementarity Persp Drug Discov Des 2000 20 171 190 10.1023/A:1008737207775

[B74] Zavodszky MI Kuhn LA Side-chain flexibility in protein-ligand binding: the minimal rotation hypothesis Protein Sci 2005 14 1104 1114 10.1110/ps.041153605 15772311 PMC2253453

[B75] Alberts IL Todorov NP Dean PM Receptor flexibility in de novo ligand design and docking J Med Chem 2005 48 6585 6596 10.1021/jm050196j 16220975

[B76] Chen HM Liu BF Huang HL Hwang SF Ho SY SODOCK: Swarm optimization for highly flexible protein-ligand docking J Comput Chem 2007 28 612 623 10.1002/jcc.20542 17186483

[B77] Fradera X Knegtel RMA Mestres J Similarity-driven flexible ligand docking Proteins Struct Funct Genet 2000 40 623 636 10.1002/1097-0134(20000901)40:4<623::AID-PROT70>3.0.CO;2-I 10899786

[B78] Jain AN Surflex: fully automatic flexible molecular docking using a molecular similarity-based search engine J Med Chem 2003 46 499 511 10.1021/jm020406h 12570372

[B79] Jain AN Surflex-Dock 2.1: robust performance from ligand energetic modeling, ring flexibility, and knowledge-based search J Comput Aided Mol Des 2007 21 281 306 10.1007/s10822-007-9114-2 17387436

[B80] Choi V YUCCA: an efficient algorithm for small-molecule docking Chem Biodivers 2005 2 1517 1524 10.1002/cbdv.200590123 17191951

[B81] Khanna V Ranganathan S In silico approach to screen compounds activeagainst parasitic nematodes of major socioeconomic importance BMC Bioinformatics 2011 12 Suppl 13 S25 10.1186/1471-2105-12-S13-S25 22373185 PMC3278842

[B82] Rastelli G Pacchioni S Sirawaraporn W Sirawaraporn R Parenti MD Ferrari AM Docking and database screening reveal new classes of Plasmodium falciparum dihydrofolate reductase inhibitors Journal of Medicinal Chemistry 2003 46 14 2834 45 10.1021/jm030781p 12825927

[B83] Kapetanovic IM COMPUTER-AIDED DRUG DISCOVERY AND DEVELOPMENT (CADDD): in silico-chemico-biological approach Chem Biol Interact 2008 171 2 165 176 10.1016/j.cbi.2006.12.006 17229415 PMC2253724

[B84] Talele TT Khedkar SA Rigby AC Successful applications of computer aided drug discovery: moving drugs from concept to the clinic Curr Top Med Chem 2010 10 1 127 41 10.2174/156802610790232251 19929824

[B85] Ooms F Molecular modeling and computer aided drug design. Examples of their applications in medicinal chemistry Current Medicinal Chemistry 2000 7 141 158 10637360 10.2174/0929867003375317

[B86] Douglas BK Decornez H Furr JR Bajorath J Docking and scoring in virtual screening for drug discovery: methods and applications Nature Reviews Drug Discovery 2004 3 935 949 10.1038/nrd1549 15520816

[B87] Ekins S Mestres J Testa B In silico pharmacology for drug discovery: methods for virtual ligand screening and profiling Br J Pharmacol 2007 152 1 9 20 10.1038/sj.bjp.0707305 17549047 PMC1978274

[B88] Pang YP In Silico Drug Discovery: Solving the "target-rich and lead-poor" imbalance using the genome-to-drug-lead paradigm Clinical Pharmacology & Therapeutics 2007 81 30 34 10.1038/sj.clpt.6100030 17185996 PMC7162381

[B89] Rao VS Srinivas K Modern drug discovery process: an in silico approach Journal of Bioinformatics and Sequence Analysis 2011 2 5 89 94

[B90] Singh Tanya Biswas D Jayaram B A robust active site identification protocol based on physico-chemical descriptors lining the cavities in proteins J Chem Inf Modeling 2011 51 10 2515 2527 10.1021/ci200193z 21877713

[B91] Irwin JJ Shoichet BK ZINC - free database of commercially available compounds for virtual screening J Chem Inf Model 2005 45 177 182 10.1021/ci049714+ 15667143 PMC1360656

[B92] Jakalian A Bush BL Jack DB Bayly CI Fast, efficient generation of high-quality atomic charges. AM1-BCC model: I. Method J Comput Chem 2000 21 132 146 10.1002/(SICI)1096-987X(20000130)21:2<132::AID-JCC5>3.0.CO;2-P 12395429

[B93] Mukherjee G Patra N Barua P Jayaram B A fast empirical GAFF compatible partial atomic charge assignment scheme for modeling interactions of small molecules with biomolecular targets J Comput Chem 2011 32 893 907 10.1002/jcc.21671 21341292

[B94] Wang J Wolf RM Caldwell JW Kollman PA Case DA Development and testing of a general amber force field J Comput Chem 2004 25 1157 1174 10.1002/jcc.20035 15116359

[B95] Cornell WD Cieplak P Bayly CI Gould IR Merz KM A second generation force field for the simulation of proteins, nucleic acids, and organic molecules J Am Chem Soc 1995 117 5179 5197 10.1021/ja00124a002

[B96] Gupta A Gandhimathi P Sharma P Jayaram B ParDOCK: An all atom energy based Monte Carlo docking protocol for protein-ligand complexes Protein Pept Lett 2007 14 632 46 17897088 10.2174/092986607781483831

[B97] Jain T Jayaram B An all atom energy based computational protocol for predicting binding affinities of protein-ligand complexes FEBS Letters 2005 579 6659 6666 10.1016/j.febslet.2005.10.031 16307743

[B98] Jain T Jayaram B Computational protocol for predicting the binding affinities of Zinc containing metalloprotein-ligand complexes PROTEINS: Struct Funct Bioinfo 2007 67 1167 1178 10.1002/prot.21332 17380508

[B99] Shaikh S Jayaram B A swift all atom energy based computational protocol to predict DNA-Drug binding affinity and ΔTm J Med Chem 2007 50 2240 2244 10.1021/jm060542c 17419602

[B100] Shaikh SA Ahmed SR Jayaram B A molecular thermodynamic view of DNA-drug interaction: a case study of 25 minor groove binders Arch Biochem Biophys 2004 429 81 99 10.1016/j.abb.2004.05.019 15288812

[B101] Kalra P Reddy V Jayaram B A free energy component analysis of HIV-I protease - inhibitor binding J Med Chem 2001 44 4325 4338 10.1021/jm010175z 11728180

[B102] Lipinski CA Lead- and drug-like compounds: the rule-of-five revolution Drug Discovery Today: Technologies 2004 1 337 341 10.1016/j.ddtec.2004.11.007 24981612

[B103] Lipinski CA Lombardo F Dominy BW Feeney PJ Experimental and computational approaches to estimate solubility and permeability in drug discovery and development settings Adv Drug Delivery Rev 1997 23 3 25 10.1016/S0169-409X(96)00423-1 11259830

[B104] Berman HM Westbrook J Feng Z Gilliland G Bhat TN Weissig H Shindyalov IN Bourne PE The Protein Data Bank Nucleic Acids Res 2000 28 235 242 10.1093/nar/28.1.235 10592235 PMC102472

[B105] Pearlman DA Case DA Caldwell JW Ross WS Cheathem JE III AMBER, a package of computer programs for applying molecular mechanics, normal mode analysis, molecular dynamics and free energy calculations to simulate the structural and energetic properties of molecules Comput Phys Commun 1995 91 1 41 10.1016/0010-4655(95)00041-D

[B106] Zhang C Liu S Zhu Q Zhou Y A knowledge based energy function for protein-ligand, protein-protein and protein-DNA complexes J Med Chem 2005 48 2325 2335 10.1021/jm049314d 15801826

[B107] Wang R Lai L Wang S Further development and validation of empirical scoring functions for structure-based binding affinity prediction J Comput Aided Mol Des 2002 16 11 26 10.1023/A:1016357811882 12197663

[B108] DeWitte RS Shakhnovich EI SMoG: de novo design method based on simple, fast and accurate free energy estimates. Methodology and supporting evidence J Am Chem Soc 1996 118 11733 11744 10.1021/ja960751u

[B109] Mitchell JBO Laskowski RA Alex A Thornton JM BLEEP: potential of mean force describing protein-ligand interactions: II. Calculation of binding energies and comparison with experimental data J Comp Chem 1999 20 1177 1185 10.1002/(SICI)1096-987X(199908)20:11<1177::AID-JCC8>3.0.CO;2-0

[B110] Muegge I Martin YC A general and fast scoring function for protein-ligand interactions: a simplified potential approach J Med Chem 1999 42 791 804 10.1021/jm980536j 10072678

[B111] Wang R Liu L Lai L Tang Y SCORE: a new empirical method for estimating the binding affinity of a protein-ligand complex J Mol Model 1998 4 379 394 10.1007/s008940050096

[B112] Bohm HJ Prediction of binding constants of protein-ligands: a fast method for the prioritization of hits obtained from de novo design or 3D database search programs J Comput Aided Mol Des 1998 12 309 323 10.1023/A:1007999920146 9777490

[B113] Eldridge MD Murray CW Auton TR Paolini GV Mee RP Empirical scoring functions: I. The development of a fast empirical scoring function to estimate the binding affinity of ligands in receptor complexes J Comput Aided Mol Des 1997 11 425 445 10.1023/A:1007996124545 9385547

[B114] Krammer A Kirchhoff PD Jiang X Venkatachalam CM Waldman M LigScore: a novel scoring function for predicting binding affinities J Mol Graph Model 2005 23 395 407 10.1016/j.jmgm.2004.11.007 15781182

[B115] Cheng T Liu Z Wang R A knowledge-guided strategy for improving the accuracy of scoring functions in binding affinity prediction BMC Bioinformatics 2010 11 193 10.1186/1471-2105-11-193 20398404 PMC2868011

[B116] Zhao X Liu X Wang Y Chen Z Kang L Zhang H Luo X Zhu W Chen K Li H Wang X Jiang H An improved PMF scoring function for universally predicting the interactions of a ligand with protein, DNA, and RNA J Chem Inf Model 2008 48 7 1438 47 10.1021/ci7004719 18553962

[B117] Raha K Merz KM Jr A quantum mechanics based scoring function: study of zinc ion-mediated ligand binding J Am Chem Soc 2004 126 1020 1021 10.1021/ja038496i 14746460

[B118] Hou T Zhang W Xu XJ Binding affinities for a series of selective inhibitors of gelatinase-A using molecular dynamics with a linear interaction energy approach J Phys Chem B 2001 105 5304 5315

[B119] Hu X Shelver WH Docking studies of matrix metalloproteinase inhibitors: zinc parameter optimization to improve the binding free energy prediction J Mol Graph Model 2003 22 115 126 10.1016/S1093-3263(03)00153-0 12932782

[B120] Rizzo RC Toba S Kuntz ID A molecular basis for the selectivity of thiadiazole urea inhibitors with stromelysin-1 and gelatinase-A from generalized born molecular dynamics simulations J Med Chem 2004 47 3065 3074 10.1021/jm030570k 15163188

[B121] Khandelwal A Lukacova V Comez D Kroll DM Raha S Balaz S A combination of docking, QM/MM methods, and MD simulation for binding affinity estimation of metalloprotein ligands J Med Chem 2005 48 5437 5447 10.1021/jm049050v 16107143 PMC2896055

[B122] Xie ZR Hwang MJ An interaction-motif-based scoring function for protein-ligand docking BMC Bioinformatics 2010 11 298 10.1186/1471-2105-11-298 20525216 PMC3098071

[B123] Arnott S Campbell-Smith PJ Chandrasekaran R Fasman GP In handbook of biochemistry and molecular biology Nucleic Acids--Volume II 1976 3 Cleveland: CRC Press 411 422

[B124] Beveridge DL Barreiro G Byun KS Case DA Cheatham TE Dixit SB Giudice E Lankas F Lavery R Maddocks JH Osman R Seibert E Sklenar H Stoll G Thayer KM Varnai P Young MA Molecular dynamics simulations of the 136 unique tetranucleotide sequences of DNA oligonucleotides. I. research design and results on d(CpG) steps Biophysical Journal 2004 87 6 3799 3813 10.1529/biophysj.104.045252 15326025 PMC1304892

[B125] Lavery R Zakrzewska K Beveridge DL Bishop TC Case DA Cheatham T III Dixit S Jayaram B Lankas F Laughton C Maddocks JH Michon A Osman R Orozco M Perez A Singh T Spackova N Sponer J A systematic molecular dynamics study of nearest neighbor effects on base pair and base pair step conformations and fluctuations in B-DNA Nucleic Acids Research 2009 38 1 299 313 19850719 10.1093/nar/gkp834 PMC2800215

[B126] Kinnings SL Xie L Fung KH Jackson RM Xie L Bourne PE The Mycobacterium tuberculosis Drugome and Its Polypharmacological Implications PLoS Comput Biol 2010 6 11 10.1371/journal.pcbi.1000976 PMC2973814 21079673

[B127] Antalis TM Buzza MS Hodge KM Hooper JD Netzel-arnett S The cutting edge: membrane-anchored serine protease activities in the pericellular microenvironment Biochem J 2010 428 325 346 10.1042/BJ20100046 20507279 PMC3680374

[B128] Li L Dantzer JJ Nowacki J O'Callaghan BJ Meroueh SO PDBcal: a comprehensive dataset for receptor-ligand interactions with three-dimensional structures and binding thermodynamics from isothermal titration calorimetry Chem Biol Drug Des 2008 71 529 532 10.1111/j.1747-0285.2008.00661.x 18482338

[B129] Talhout R Engberts BFN Thermodynamic analysis of binding of p-substituted benzamidines to trypsin Eur J Biochem 2001 268 1554 1560 10.1046/j.1432-1327.2001.01991.x 11248672

[B130] Soni A Menaria K Ray P Jayaram B Genomes to hits *in Silico *- a country path today, a highway tomorrow: a case study of Chikungunya Current Pharmaceutical Design 2012 accepted for publication 10.2174/13816128113199990379 PMC3831887 23260020

