# Supplementary material for: Sanjeevini: a freely accessible web-server for target directed lead molecule discovery
Source: BMC Bioinformatics. 2012 Dec 7;13(Suppl 17):S7. doi: 10.1186/1471-2105-13-S17-S7 (PMC3521208; doi:10.1186/1471-2105-13-S17-S7)
Supplement: Additional file 1 — Validation of Sanjeevini scoring function on 366 Protein/DNA-ligand complexes. [file 1471-2105-13-S17-S7-S1.docx]

**Additional file 1**: Validation of *Sanjeevini* scoring function on 366 Protein/DNA-ligand complexes

| **Sl.No** | **PDBID^a^** | **Drug Target** | **EBFE^b^** | **PBFE^c^** |
| --- | --- | --- | --- | --- |
| 1 | 127D | DNA | -11 | -11.2 |
| 2 | 264D | DNA | -11.7 | -12 |
| 3 | 109D | DNA | -12 | -11.7 |
| 4 | 2DBE | DNA | -8.6 | -8.5 |
| 5 | 1D63 | DNA | -8 | -9 |
| 6 | 261D | DNA | -11.5 | -11.5 |
| 7 | 1JTLM | DNA | -11.5 | -12 |
| 8 | 227D | DNA | -9.3 | -8.5 |
| 9 | 1PRP | DNA | -8.2 | -9.4 |
| 10 | 2GXI | DNA | -8.5 | -9.4 |
| 11 | 2GXJ | DNA | -9.1 | -8.3 |
| 12 | 2GXK | DNA | -9 | -8.7 |
| 13 | 2IRJ | DNA | -8.6 | -8.6 |
| 14 | 2GXM | DNA | -8.8 | -8.8 |
| 15 | 2GXN | DNA | -9.2 | -8.7 |
| 16 | 2GXO | DNA | -9.1 | -8.3 |
| 17 | 2GXP | DNA | -9.1 | -8.8 |
| 18 | 2GXR | DNA | -10.7 | -10.2 |
| 19 | 2GXT | DNA | -10.9 | -10.8 |
| 20 | 2GXV | DNA | -11.9 | -9.8 |
| 21 | 2IRK | DNA | -8.1 | -7.6 |
| 22 | 2GXX | DNA | -8.1 | -7.9 |
| 23 | 2GXY | DNA | -10.7 | -9.3 |
| 24 | 2IRL | DNA | -10.8 | -10.6 |
| 25 | 2GY0 | DNA | -9.7 | -8.9 |
| 26 | 2GY1 | DNA | -9.2 | -9 |
| 27 | 2GY2 | DNA | -12.7 | -12.3 |
| 28 | 2GY3 | DNA | -11.8 | -12.3 |
| 29 | 2GY4 | DNA | -11.6 | -11.7 |
| 30 | 2GY6 | DNA | -11.9 | -10.8 |
| 31 | 2GY8 | DNA | -11.9 | -10.9 |
| 32 | 2GYE | DNA | -11.9 | -11.1 |
| 33 | 2GYF | DNA | -12.1 | -11.8 |
| 34 | 2GYG | DNA | -12.3 | -12.1 |
| 35 | 2GYH | DNA | -12.3 | -11.5 |
| 36 | 2GYJ | DNA | -13 | -12.6 |
| 37 | 2GYL | DNA | -13.1 | -12.9 |
| 38 | 2GYM | DNA | -9.4 | -9.9 |
| 39 | 2GYN | DNA | -8.1 | -9.6 |
| 40 | 1A30 | Protein | -5.84 | -5.78 |
| 41 | 1A4K | Protein | -6.79 | -7.21 |
| 42 | 1A4W | Protein | -8.13 | -10.06 |
| 43 | 1A9M | Protein | -9.41 | -10.33 |
| 44 | 1AAQ | Protein | -11.45 | -11.17 |
| 45 | 1AE8 | Protein | -8.99 | -8.18 |
| 46 | 1AFK | Protein | -9 | -11.37 |
| 47 | 1AFL | Protein | -8.53 | -7.74 |
| 48 | 1AJV | Protein | -10.59 | -10.81 |
| 49 | 1AJX | Protein | -10.86 | -10.19 |
| 50 | 1ANF | Protein | -7.442 | -5.83 |
| 51 | 1APT | Protein | -12.82 | -11.29 |
| 52 | 1APU | Protein | -10.5 | -10.34 |
| 53 | 1APV | Protein | -12.35 | -11.4 |
| 54 | 1APW | Protein | -10.98 | -9.31 |
| 55 | 1B38 | Protein | -9.4 | -10.64 |
| 56 | 1B39 | Protein | -8.97 | -9.26 |
| 57 | 1B5G | Protein | -10.911 | -10.51 |
| 58 | 1B6J | Protein | -10.76 | -12.05 |
| 59 | 1B6K | Protein | -11.88 | -12.69 |
| 60 | 1B6L | Protein | -11.28 | -11.02 |
| 61 | 1B6M | Protein | -11.41 | -11.9 |
| 62 | 1BA8 | Protein | -12.27 | -10.27 |
| 63 | 1BB0 | Protein | -11.4 | -10.14 |
| 64 | 1BDR | Protein | -9.07 | -10.51 |
| 65 | 1BIL | Protein | -12.64 | -12.86 |
| 66 | 1BIM | Protein | -12.12 | -13.88 |
| 67 | 1BMM | Protein | -9.75 | -10.08 |
| 68 | 1BMN | Protein | -11.58 | -9.06 |
| 69 | 1BRA | Protein | -2.496 | -4.83 |
| 70 | 1BV7 | Protein | -12.64 | -14.84 |
| 71 | 1C2T | Protein | -11.066 | -10.89 |
| 72 | 1C5C | Protein | -9.49 | -7.81 |
| 73 | 1C83 | Protein | -6.59 | -5.62 |
| 74 | 1C84 | Protein | -6.79 | -8.97 |
| 75 | 1C86 | Protein | -7.09 | -8.45 |
| 76 | 1C87 | Protein | -6.59 | -8.4 |
| 77 | 1C88 | Protein | -8.86 | -8.96 |
| 78 | 1C8K | Protein | -6.79 | -7.7 |
| 79 | 1CBS | Protein | -9.821 | -7.49 |
| 80 | 1CF8 | Protein | -8.226 | -8.14 |
| 81 | 1COM | Protein | -5.443 | -6.38 |
| 82 | 1CQP | Protein | -6.614 | -6.41 |
| 83 | 1CTR | Protein | -5.845 | -5.52 |
| 84 | 1D3H | Protein | -6.008 | -7.29 |
| 85 | 1D3P | Protein | -10.07 | -11.11 |
| 86 | 1D4L | Protein | -11.92 | -12.19 |
| 87 | 1D4P | Protein | -8.594 | -8.76 |
| 88 | 1DG5 | Protein | -5.532 | -6.66 |
| 89 | 1DHF | Protein | -10.093 | -9.47 |
| 90 | 1DMP | Protein | -13.029 | -12.13 |
| 91 | 1DOG | Protein | -5.479 | -3.02 |
| 92 | 1DR1 | Protein | -7.597 | -5.28 |
| 93 | 1DRF | Protein | -10.148 | -9.02 |
| 94 | 1DWB | Protein | -3.983 | -4.78 |
| 95 | 1DWC | Protein | -10.105 | -8.55 |
| 96 | 1DWD | Protein | -11.138 | -9.79 |
| 97 | 1DY9 | Protein | -10.323 | -9.93 |
| 98 | 1EAP | Protein | -8.468 | -8.39 |
| 99 | 1ELA | Protein | -8.66 | -8.64 |
| 100 | 1ELC | Protein | -9.76 | -9.08 |
| 101 | 1EPO | Protein | -10.856 | -9.74 |
| 102 | 1EPP | Protein | -9.77 | -9.66 |
| 103 | 1ETR | Protein | -10.108 | -9.45 |
| 104 | 1ETS | Protein | -11.623 | -9.28 |
| 105 | 1ETT | Protein | -8.441 | -8.81 |
| 106 | 1FAX | Protein | -10.074 | -10.07 |
| 107 | 1FKG | Protein | -8.812 | -6.7 |
| 108 | 1FLR | Protein | -6.347 | -6.31 |
| 109 | 1G2K | Protein | -10.81 | -11.64 |
| 110 | 1GNO | Protein | -10.57 | -9.85 |
| 111 | 1GPY | Protein | -6.409 | -6.29 |
| 112 | 1HBV | Protein | -8.688 | -10.29 |
| 113 | 1HDT | Protein | -10.66 | -10.94 |
| 114 | 1HEW | Protein | -8.183 | -8.55 |
| 115 | 1HGE | Protein | -3.093 | -4.17 |
| 116 | 1HGH | Protein | -3.89 | -4.79 |
| 117 | 1HGI | Protein | -3.652 | -4.64 |
| 118 | 1HGJ | Protein | -2.262 | -3.57 |
| 119 | 1HIH | Protein | -10.97 | -11.76 |
| 120 | 1HII | Protein | -9.89 | -11.75 |
| 121 | 1HIV | Protein | -12.64 | -13.03 |
| 122 | 1HOS | Protein | -11.74 | -11.98 |
| 123 | 1HPO | Protein | -11.82 | -11.37 |
| 124 | 1HPS | Protein | -12.66 | -10.64 |
| 125 | 1HPV | Protein | -12.584 | -10.57 |
| 126 | 1HPX | Protein | -12.53 | -12.97 |
| 127 | 1HRI | Protein | -5.914 | -7.61 |
| 128 | 1HRN | Protein | -10.94 | -11.95 |
| 129 | 1HSG | Protein | -12.93 | -12.56 |
| 130 | 1HSH | Protein | -11.69 | -12.13 |
| 131 | 1HTE | Protein | -7.69 | -7.69 |
| 132 | 1HTG | Protein | -13.215 | -12.52 |
| 133 | 1HVH | Protein | -10.81 | -10.54 |
| 134 | 1HVI | Protein | -13.748 | -15.66 |
| 135 | 1HVJ | Protein | -14.267 | -14.72 |
| 136 | 1HVK | Protein | -13.803 | -15.46 |
| 137 | 1HVL | Protein | -12.289 | -14.72 |
| 138 | 1HVR | Protein | -12.972 | -12.92 |
| 139 | 1HXB | Protein | -13.48 | -12.52 |
| 140 | 1HXW | Protein | -14.54 | -14.34 |
| 141 | 1IDA | Protein | -11.865 | -12.41 |
| 142 | 1LYB | Protein | -15.575 | -12.04 |
| 143 | 1MCB | Protein | -6.6 | -8.38 |
| 144 | 1MCF | Protein | -7.02 | -8.57 |
| 145 | 1MCH | Protein | -7.02 | -8.75 |
| 146 | 1MCJ | Protein | -5.16 | -5.74 |
| 146 | 1MCS | Protein | -6.6 | -8.14 |
| 147 | 1MTW | Protein | -10.076 | -8.06 |
| 148 | 1NNB | Protein | -5.458 | -7.04 |
| 149 | 1NSD | Protein | -7.23 | -7.52 |
| 150 | 1OLA | Protein | -9.546 | -10.25 |
| 151 | 1PGP | Protein | -7.77 | -6.49 |
| 152 | 1PHH | Protein | -3.983 | -4.41 |
| 153 | 1PPC | Protein | -8.8 | -8.38 |
| 154 | 1PPH | Protein | -8.487 | -6.62 |
| 155 | 1PPM | Protein | -7.95 | -9.53 |
| 156 | 1QBR | Protein | -14.42 | -14.54 |
| 157 | 1QBT | Protein | -14.492 | -14.36 |
| 158 | 1QBU | Protein | -13.968 | -11.9 |
| 159 | 1RBP | Protein | -9.17 | -7.94 |
| 160 | 1RGK | Protein | -5.879 | -6.88 |
| 161 | 1RGL | Protein | -6.041 | -5.97 |
| 162 | 1SRE | Protein | -5.455 | -6.27 |
| 163 | 1TLC | Protein | -10.98 | -9.75 |
| 164 | 1TMT | Protein | -8.51 | -7.9 |
| 165 | 1TNG | Protein | -4 | -3.91 |
| 166 | 1TNH | Protein | -4.59 | -4.3 |
| 167 | 1TNI | Protein | -2.32 | -3.47 |
| 168 | 1TNJ | Protein | -2.67 | -3.63 |
| 169 | 1TNK | Protein | -2.03 | -3.63 |
| 170 | 1TNL | Protein | -2.56 | -4.21 |
| 171 | 1TPH | Protein | -3.098 | -5.54 |
| 172 | 1TPP | Protein | -7.95 | -6.27 |
| 173 | 1ULB | Protein | -2.802 | -3.19 |
| 174 | 1UVS | Protein | -7.41 | -7.49 |
| 175 | 2ABH | Protein | -8.876 | -7.64 |
| 176 | 2ACS | Protein | -2.75 | -4.86 |
| 177 | 2CGR | Protein | -9.928 | -10.66 |
| 178 | 2CMD | Protein | -6.24 | -7.68 |
| 179 | 2IFB | Protein | -7.406 | -8.28 |
| 180 | 2MSB | Protein | -3.937 | -2.35 |
| 181 | 2UPJ | Protein | -10.14 | -10.41 |
| 182 | 2WEA | Protein | -8.37 | -6.59 |
| 183 | 2WEB | Protein | -7.03 | -8.5 |
| 184 | 2WEC | Protein | -6.8 | -6.53 |
| 185 | 2YPI | Protein | -6.58 | -7.04 |
| 186 | 3CLA | Protein | -6.037 | -3.92 |
| 187 | 3PTB | Protein | -6.46 | -5.39 |
| 188 | 4DFR | Protein | -13.23 | -11.23 |
| 189 | 4EST | Protein | -9.551 | -7.8 |
| 190 | 4HMG | Protein | -3.481 | -3.96 |
| 191 | 4PHV | Protein | -12.479 | -12 |
| 192 | 4TS1 | Protein | -7.64 | -4.96 |
| 193 | 5CNA | Protein | -2.73 | -3.32 |
| 194 | 5HVP | Protein | -10.516 | -12.79 |
| 195 | 5TIM | Protein | -3.14 | -3.07 |
| 196 | 7DFR | Protein | -10.091 | -10.3 |
| 197 | 7GPB | Protein | -7.533 | -7.87 |
| 198 | 8GPB | Protein | -4.913 | -5.97 |
| 199 | 1BNM | MetalloProteinase | -13.64 | -11.81 |
| 200 | 1BNN | MetalloProteinase | -13.64 | -12.74 |
| 201 | 1I8Z | MetalloProteinase | -13.57 | -13.62 |
| 202 | 1I91 | MetalloProteinase | -13.57 | -14.76 |
| 203 | 1BN3 | MetalloProteinase | -13.49 | -12.8 |
| 204 | 1BNT | MetalloProteinase | -13.37 | -12.81 |
| 205 | 1IF8 | MetalloProteinase | -13.32 | -12.06 |
| 206 | 1BNU | MetalloProteinase | -13.23 | -13.13 |
| 207 | 1BNQ | MetalloProteinase | -12.94 | -11.97 |
| 208 | 1CIL | MetalloProteinase | -12.94 | -12.94 |
| 209 | 1BN1 | MetalloProteinase | -12.82 | -11.29 |
| 210 | 1BN4 | MetalloProteinase | -12.7 | -11.26 |
| 211 | 1OKL | MetalloProteinase | -8.23 | -7.69 |
| 212 | 1BNW | MetalloProteinase | -12.38 | -11.22 |
| 213 | 1CIM | MetalloProteinase | -12.1 | -11.42 |
| 214 | 1CIN | MetalloProteinase | -11.97 | -11.32 |
| 215 | 1BNV | MetalloProteinase | -11.96 | -12.65 |
| 216 | 1I9N | MetalloProteinase | -11.96 | -11.2 |
| 217 | 2H4N | MetalloProteinase | -11.87 | -9.52 |
| 218 | 1I9L | MetalloProteinase | -11.72 | -11.01 |
| 219 | 1I9M | MetalloProteinase | -11.72 | -10.31 |
| 220 | 1I9O | MetalloProteinase | -11.63 | -11.75 |
| 221 | 1I9Q | MetalloProteinase | -11.62 | -10.37 |
| 222 | 1CNY | MetalloProteinase | -10.78 | -9.15 |
| 223 | 1OQ5 | MetalloProteinase | -10.61 | -11.01 |
| 224 | 1CNW | MetalloProteinase | -10.6 | -11.33 |
| 225 | 1CNX | MetalloProteinase | -10.11 | -8.85 |
| 226 | 1EOU | MetalloProteinase | -9.78 | -11.87 |
| 227 | 1AZM | MetalloProteinase | -8.35 | -7.99 |
| 228 | 1BZM | MetalloProteinase | -8.23 | -10.06 |
| 229 | 1OKM | MetalloProteinase | -7.93 | -9.1 |
| 230 | 1AM6 | MetalloProteinase | -5.9 | -6.42 |
| 231 | 1AVN | MetalloProteinase | -2.88 | -3.15 |
| 232 | 1BCD | MetalloProteinase | -5.32 | -6.53 |
| 233 | 6CPA | MetalloProteinase | -15.71 | -15.58 |
| 234 | 1CPS | MetalloProteinase | -9.08 | -8.13 |
| 235 | 1CBX | MetalloProteinase | -8.65 | -7.5 |
| 236 | 2CTC | MetalloProteinase | -5.31 | -6.28 |
| 237 | 3CPA | MetalloProteinase | -5.3 | -6.51 |
| 238 | 1G4K | MetalloProteinase | -7.87 | -8.24 |
| 239 | 1MMR | MetalloProteinase | -8.03 | -8.39 |
| 240 | 1JJ9 | MetalloProteinase | -7.97 | -7.47 |
| 241 | 1B8Y | MetalloProteinase | -10.85 | -10.13 |
| 242 | 1CAQ | MetalloProteinase | -10.67 | -10.76 |
| 243 | 1CIZ | MetalloProteinase | -10.28 | -10.38 |
| 244 | 1SLN | MetalloProteinase | -9.06 | -9.39 |
| 245 | 1MMP | MetalloProteinase | -8.51 | -8.98 |
| 246 | 1HY7 | MetalloProteinase | -8.44 | -7.5 |
| 247 | 1C3I | MetalloProteinase | -8.01 | -9.92 |
| 248 | 1C8T | MetalloProteinase | -8.01 | -8.56 |
| 249 | 1JAO | MetalloProteinase | -6.8 | -6.22 |
| 250 | 1MMB | MetalloProteinase | -12.58 | -10.99 |
| 251 | 2TCL | MetalloProteinase | -11.12 | -11.74 |
| 252 | 966C | MetalloProteinase | -10.56 | -10.93 |
| 253 | 456C | MetalloProteinase | -13.5 | -12.13 |
| 254 | 830C | MetalloProteinase | -12.82 | -12.84 |
| 255 | 1D8M | MetalloProteinase | -11.76 | -10.35 |
| 256 | 1G05 | MetalloProteinase | -11.76 | -12.3 |
| 257 | 1BQO | MetalloProteinase | -10.7 | -12.41 |
| 258 | 1D8F | MetalloProteinase | -10.7 | -9.95 |
| 259 | 1B3D | MetalloProteinase | -10.52 | -10.85 |
| 260 | 1D5J | MetalloProteinase | -12.65 | -10.14 |
| 261 | 1G49 | MetalloProteinase | -10.77 | -8.93 |
| 262 | 1MMQ | MetalloProteinase | -12.28 | -11.29 |
| 263 | 1MNC | MetalloProteinase | -12.27 | -9.49 |
| 264 | 1A85 | MetalloProteinase | -10.49 | -10.61 |
| 265 | 1A86 | MetalloProteinase | -5.52 | -5.2 |
| 266 | 1JAP | MetalloProteinase | -6.52 | -8.74 |
| 267 | 1-JAN | MetalloProteinase | -6.52 | -8.31 |
| 268 | 1JAQ | MetalloProteinase | -6.19 | -6.87 |
| 269 | 1CXV | MetalloProteinase | -13.5 | -11.59 |
| 270 | 1QF0 | MetalloProteinase | -10.19 | -10.02 |
| 271 | 1THL | MetalloProteinase | -8.76 | -8.15 |
| 272 | 1TMN | MetalloProteinase | -9.95 | -8.68 |
| 273 | 3TMN | MetalloProteinase | -8.04 | -6.89 |
| 274 | 4TMN | MetalloProteinase | -13.89 | -13.23 |
| 275 | 5TMN | MetalloProteinase | -10.96 | -10.3 |
| 276 | 6TMN | MetalloProteinase | -6.88 | -8.54 |
| 277 | 1TLP | MetalloProteinase | -10.44 | -8.97 |
| 278 | 2TMN | MetalloProteinase | -8.03 | -7.09 |
| 279 | 1QF1 | MetalloProteinase | -10.11 | -10.56 |
| 280 | 1QF2 | MetalloProteinase | -8.18 | -9.31 |
| 281 | 1LDY | MetalloProteinase | -11.06 | -10.15 |
| 282 | 1LDE | MetalloProteinase | -9.41 | -10.11 |
| 283 | 1BTO | MetalloProteinase | -8.93 | -7.92 |
| 284 | 3BTO | MetalloProteinase | -8.43 | -7.8 |
| 285 | 1HLD | MetalloProteinase | -7.58 | -7.26 |
| 286 | 1A4Q | Protein | -11.77 | -9.13 |
| 287 | 1AC4 | Protein | -3.85 | -3.94 |
| 288 | 1ACM | Protein | -10.31 | -8.14 |
| 289 | 1ACO | Protein | -5.17 | -8.49 |
| 290 | 1AEB | Protein | -4.81 | -3.15 |
| 291 | 1AEE | Protein | -3.96 | -3.15 |
| 292 | 1AI4 | Protein | -3.46 | -3.98 |
| 293 | 1AI5 | Protein | -5.14 | -4.27 |
| 294 | 1AI6 | Protein | -5.46 | -4.7 |
| 295 | 1AJN | Protein | -3.64 | -4.22 |
| 296 | 1AJP | Protein | -3.08 | -4 |
| 297 | 1APB | Protein | -7.94 | -4.11 |
| 298 | 1B9S | Protein | -4.39 | -6.48 |
| 299 | 1B9T | Protein | -7.04 | -8.2 |
| 300 | 1B9V | Protein | -5.04 | -9.27 |
| 301 | 1BV9 | Protein | -12.17 | -14.04 |
| 302 | 1BWA | Protein | -10.33 | -14.2 |
| 303 | 1C29 | Protein | -8.74 | -5.81 |
| 304 | 1C85 | Protein | -6.41 | -8.26 |
| 305 | 1C9D | Protein | -8.71 | -8.81 |
| 306 | 1CDG | Protein | -3.3 | -2.47 |
| 307 | 1CPI | Protein | -10.07 | -12.97 |
| 308 | 1CSC | Protein | -9.81 | -12.41 |
| 309 | 1CTT | Protein | -6.24 | -3.86 |
| 310 | 1CVU | Protein | -10.76 | -6.93 |
| 311 | 1CW2 | Protein | -8.49 | -8.09 |
| 312 | 1CX2 | Protein | -10.94 | -7.06 |
| 313 | 1CX9 | Protein | -9.32 | -7.69 |
| 314 | 1D3D | Protein | -12.55 | -10.59 |
| 315 | 1D3T | Protein | -7.78 | -9.76 |
| 316 | 1DIE | Protein | -2.92 | -2.56 |
| 317 | 1EED | Protein | -6.54 | -8.99 |
| 318 | 1ELB | Protein | -9.75 | -7.04 |
| 319 | 1ELD | Protein | -9.13 | -8.83 |
| 320 | 1ELE | Protein | -9.33 | -9.06 |
| 321 | 1ENT | Protein | -9.55 | -8.37 |
| 322 | 1ENU | Protein | -7.02 | -5.89 |
| 323 | 1EZQ | Protein | -12.5 | -11.34 |
| 324 | 1F0R | Protein | -10.58 | -8.77 |
| 325 | 1F0T | Protein | -8.29 | -8.24 |
| 326 | 1F0U | Protein | -9.89 | -9.68 |
| 327 | 1F3E | Protein | -9.25 | -6.37 |
| 328 | 1GHB | Protein | -1.71 | -6.05 |
| 329 | 1HDC | Protein | -8.17 | -7.83 |
| 330 | 1HTF | Protein | -11.04 | -9.95 |
| 331 | 1IMB | Protein | -5.73 | -4.04 |
| 332 | 1IVB | Protein | -4.14 | -5.88 |
| 333 | 1IVF | Protein | -6.66 | -7.75 |
| 334 | 1K1L | Protein | -9.56 | -7.48 |
| 335 | 1K1N | Protein | -8.82 | -9.24 |
| 336 | 1MRK | Protein | -6.17 | -5.69 |
| 337 | 1NIS | Protein | -4.08 | -6.66 |
| 338 | 1NSC | Protein | -4.08 | -7.93 |
| 339 | 1PDZ | Protein | -5.03 | -5.11 |
| 340 | 1PPK | Protein | -10.44 | -8.94 |
| 341 | 1PSO | Protein | -14.11 | -12.06 |
| 342 | 1RNE | Protein | -11.94 | -14.72 |
| 343 | 1SNC | Protein | -9.25 | -11.39 |
| 344 | 2AK3 | Protein | -5.25 | -9.82 |
| 345 | 2ER7 | Protein | -12.27 | -13.96 |
| 346 | 2MCP | Protein | -7.13 | -6.72 |
| 347 | 2PK4 | Protein | -5.88 | -3.09 |
| 348 | 2R04 | Protein | -8.48 | -7.6 |
| 349 | 2SIM | Protein | -8.75 | -6.42 |
| 350 | 3ER3 | Protein | -9.68 | -12.19 |
| 351 | 3ER5 | Protein | -12.35 | -13.3 |
| 352 | 4ER2 | Protein | -11.03 | -10.39 |
| 353 | 5ENL | Protein | -5.24 | -4.83 |
| 354 | 6ENL | Protein | -4.14 | -5.3 |
| 355 | 6RNT | Protein | -8.67 | -7.31 |
| 356 | 6TIM | Protein | -4.37 | -7.56 |
| 357 | 7TIM | Protein | -7.35 | -6.91 |
| 358 | 9HVP | Protein | -11.38 | -13.83 |
| 359 | 1E2J | Protein | -7.2 | -5.78 |
| 360 | 1HXD | Protein | -9.9 | -9.05 |
| 361 | 1K1J | Protein | -10.41 | -8.55 |
| 362 | 1K1L | Protein | -9.74 | -7.56 |
| 363 | 2IKG | Protein | -8.46 | -9.14 |
| 364 | 2IKH | Protein | -7.48 | -4.72 |
| 365 | 2IKI | Protein | -10.2 | -9.14 |
| 366 | 2IKJ | Protein | -8.86 | -8.86 |

^a^Protein Data Bank ID

^b^Experimental binding free energy of the native target-ligand complex in kcal/mol.

^c^Predicted binding free energy of the native target-ligand complex in kcal/mol using *Sanjeevini* scoring function
