# Supplementary material for: Sanjeevini: a freely accessible web-server for target directed lead molecule discovery
Source: BMC Bioinformatics. 2012 Dec 7;13(Suppl 17):S7. doi: 10.1186/1471-2105-13-S17-S7 (PMC3521208; doi:10.1186/1471-2105-13-S17-S7)
Supplement: Additional file 2 — Docking and scoring studies on 335 Protein/DNA drug targets via Sanjeevini. [file 1471-2105-13-S17-S7-S2.docx]

**Additional file 2**: Docking and scoring studies on 335 Protein/DNA drug targets via *Sanjeevini*

| **Sl.No** | **PDBID^a^** | **EBFE^b^** | **PBFE^c^** | **RMSD^d^ (Å)** | **Drug Target** |
| --- | --- | --- | --- | --- | --- |
| 1 | 127d | -11 | -9.4 | 3.2 | DNA |
| 2 | 109d | -12 | -9.7 | 2.82 | DNA |
| 3 | 2dbe | -8.6 | -7.3 | 3.01 | DNA |
| 4 | 1d63 | -8 | -7.4 | 2.67 | DNA |
| 5 | 227d | -9.3 | -8.2 | 2.85 | DNA |
| 6 | 1prp | -8.2 | -7.6 | 2.12 | DNA |
| 7 | 2gxn | -9.2 | -8.1 | 2.75 | DNA |
| 8 | 2gxm | -8.8 | -8.1 | 3.3 | DNA |
| 9 | 1bnm | -13.64 | -12.93 | 1.4 | MetalloProteinase |
| 10 | 1bnn | -13.64 | -14.74 | 1.31 | MetalloProteinase |
| 11 | 1i8z | -13.57 | -15.02 | 0.44 | MetalloProteinase |
| 12 | 1i91 | -13.57 | -16.33 | 0.56 | MetalloProteinase |
| 13 | 1bn3 | -13.49 | -15.34 | 0.9 | MetalloProteinase |
| 14 | 1bnt | -13.37 | -13.81 | 0.88 | MetalloProteinase |
| 15 | 1if8 | -13.32 | -13.52 | 1.03 | MetalloProteinase |
| 16 | 1bnu | -13.23 | -14.27 | 0.94 | MetalloProteinase |
| 17 | 1bnq | -12.94 | -12.58 | 0.93 | MetalloProteinase |
| 18 | 1cil | -12.94 | -14.14 | 1.07 | MetalloProteinase |
| 19 | 1bn1 | -12.82 | -12.74 | 0.57 | MetalloProteinase |
| 20 | 1bn4 | -12.7 | -12.69 | 1.36 | MetalloProteinase |
| 21 | 1okl | -8.23 | -10.56 | 2.06 | MetalloProteinase |
| 22 | 1bnw | -12.38 | -11.93 | 0.56 | MetalloProteinase |
| 23 | 1cim | -12.1 | -12.74 | 1.43 | MetalloProteinase |
| 24 | 1cin | -11.97 | -12.74 | 1.26 | MetalloProteinase |
| 25 | 1bnv | -11.96 | -13.86 | 1.42 | MetalloProteinase |
| 26 | 1i9n | -11.96 | -12.88 | 0.59 | MetalloProteinase |
| 27 | 2h4n | -11.87 | -10.52 | 2.24 | MetalloProteinase |
| 28 | 1i9l | -11.72 | -12.3 | 0.36 | MetalloProteinase |
| 29 | 1i9m | -11.72 | -12.59 | 0.38 | MetalloProteinase |
| 30 | 1i9o | -11.63 | -8.44 | 3.48 | MetalloProteinase |
| 31 | 1i9q | -11.62 | -7.76 | 4.54 | MetalloProteinase |
| 32 | 1cny | -10.78 | -10.58 | 0.83 | MetalloProteinase |
| 33 | 1oq5 | -10.61 | -12.01 | 0.61 | MetalloProteinase |
| 34 | 1cnw | -10.6 | -14.03 | 0.89 | MetalloProteinase |
| 35 | 1cnx | -10.11 | -10.8 | 0.84 | MetalloProteinase |
| 36 | 1eou | -9.78 | -13.98 | 1.9 | MetalloProteinase |
| 37 | 1azm | -8.35 | -8.86 | 0.85 | MetalloProteinase |
| 38 | 1bzm | -8.23 | -11.14 | 0.31 | MetalloProteinase |
| 39 | 1okm | -7.93 | -10.47 | 1.16 | MetalloProteinase |
| 40 | 1am6 | -5.9 | -4.63 | 0.33 | MetalloProteinase |
| 41 | 1avn | -2.88 | -4.61 | 0.29 | MetalloProteinase |
| 42 | 1bcd | -5.32 | -7.64 | 1.82 | MetalloProteinase |
| 43 | 1cps | -9.08 | -8.77 | 2.64 | MetalloProteinase |
| 44 | 1cbx | -8.65 | -9.63 | 0.69 | MetalloProteinase |
| 45 | 2ctc | -5.31 | -7.24 | 0.32 | MetalloProteinase |
| 46 | 3cpa | -5.3 | -5.64 | 0.56 | MetalloProteinase |
| 47 | 1g4k | -7.87 | -9.92 | 0.94 | MetalloProteinase |
| 48 | 1mmr | -8.03 | -8.12 | 2.49 | MetalloProteinase |
| 49 | 1jj9 | -7.97 | -7.78 | 1.12 | MetalloProteinase |
| 50 | 1b8y | -10.85 | -16.82 | 1.38 | MetalloProteinase |
| 51 | 1caq | -10.67 | -13.65 | 1.35 | MetalloProteinase |
| 52 | 1ciz | -10.28 | -10.51 | 1.34 | MetalloProteinase |
| 53 | 1sln | -9.06 | -15.52 | 0.55 | MetalloProteinase |
| 54 | 1mmp | -8.51 | -10.78 | 2.45 | MetalloProteinase |
| 55 | 1hy7 | -8.44 | -10.31 | 4.12 | MetalloProteinase |
| 56 | 1c3i | -8.01 | -9.5 | 0.85 | MetalloProteinase |
| 57 | 1c8t | -8.01 | -9.23 | 0.92 | MetalloProteinase |
| 58 | 1jao | -6.8 | -6.47 | 0.27 | MetalloProteinase |
| 59 | 1mmb | -12.58 | -10.83 | 0.36 | MetalloProteinase |
| 60 | 2tcl | -11.12 | -12.65 | 0.42 | MetalloProteinase |
| 61 | 966c | -10.56 | -9.33 | 0.9 | MetalloProteinase |
| 62 | 456c | -13.5 | -12.89 | 0.89 | MetalloProteinase |
| 63 | 830c | -12.82 | -13.79 | 0.9 | MetalloProteinase |
| 64 | 1d8m | -11.76 | -10.56 | 1.68 | MetalloProteinase |
| 65 | 1g05 | -11.76 | -7.37 | 4.15 | MetalloProteinase |
| 66 | 1bqo | -10.7 | -12.28 | 2.17 | MetalloProteinase |
| 67 | 1d8f | -10.7 | -10.74 | 1.65 | MetalloProteinase |
| 68 | 1b3d | -10.52 | -10.78 | 0.43 | MetalloProteinase |
| 69 | 1d5j | -12.65 | -7.21 | 3.78 | MetalloProteinase |
| 70 | 1g49 | -10.77 | -7.35 | 4.24 | MetalloProteinase |
| 71 | 1mmq | -12.28 | -11.55 | 2.35 | MetalloProteinase |
| 72 | 1mnc | -12.27 | -8.25 | 4.39 | MetalloProteinase |
| 73 | 1a85 | -10.49 | -12.23 | 0.88 | MetalloProteinase |
| 74 | 1a86 | -5.52 | -8.06 | 0.58 | MetalloProteinase |
| 75 | 1jap | -6.52 | -4.04 | 3.67 | MetalloProteinase |
| 76 | 1-jan | -6.52 | -8.31 | 0.3 | MetalloProteinase |
| 77 | 1jaq | -6.19 | -9.07 | 0.56 | MetalloProteinase |
| 78 | 1cxv | -13.5 | -13.39 | 0.87 | MetalloProteinase |
| 79 | 1thl | -8.76 | -11.74 | 1.32 | MetalloProteinase |
| 80 | 1tmn | -9.95 | -10.43 | 0.46 | MetalloProteinase |
| 81 | 3tmn | -8.04 | -8.29 | 0.27 | MetalloProteinase |
| 82 | 4tmn | -13.89 | -14.05 | 0.37 | MetalloProteinase |
| 83 | 5tmn | -10.96 | -9.06 | 0.46 | MetalloProteinase |
| 84 | 6tmn | -6.88 | -9.53 | 0.19 | MetalloProteinase |
| 85 | 1tlp | -10.44 | -15.54 | 1.3 | MetalloProteinase |
| 86 | 2tmn | -8.03 | -12.81 | 0.25 | MetalloProteinase |
| 87 | 1qf1 | -10.11 | -11.6 | 0.28 | MetalloProteinase |
| 88 | 1qf2 | -8.18 | -11.42 | 0.9 | MetalloProteinase |
| 89 | 1ldy | -11.06 | -10.51 | 1.15 | MetalloProteinase |
| 90 | 1lde | -9.41 | -10.27 | 2.68 | MetalloProteinase |
| 91 | 1bto | -8.93 | -9.63 | 1.25 | MetalloProteinase |
| 92 | 3bto | -8.43 | -9.51 | 0.61 | MetalloProteinase |
| 93 | 1hld | -7.58 | -9.7 | 1.49 | MetalloProteinase |
| 94 | 1a30 | -5.84 | -6.22 | 1.03 | Protein |
| 95 | 1a4k | -6.79 | -8.1 | 0.63 | Protein |
| 96 | 1a4q | -11.77 | -8.99 | 0.35 | Protein |
| 97 | 1a4w | -8.13 | -9.81 | 0.44 | Protein |
| 98 | 1a9m | -9.41 | -11.32 | 0.7 | Protein |
| 99 | 1aaq | -11.45 | -11.21 | 0.27 | Protein |
| 100 | 1ac4 | -3.85 | -3.92 | 0.57 | Protein |
| 101 | 1acm | -10.31 | -7.54 | 3.1 | Protein |
| 102 | 1aco | -5.17 | -8.32 | 1.18 | Protein |
| 103 | 1ae8 | -8.99 | -8.26 | 0.37 | Protein |
| 104 | 1aeb | -4.81 | -3.46 | 2.94 | Protein |
| 105 | 1aee | -3.96 | -3.09 | 1.41 | Protein |
| 106 | 1ai4 | -3.46 | -4.09 | 0.49 | Protein |
| 107 | 1ai5 | -5.14 | -4.57 | 0.17 | Protein |
| 108 | 1ai6 | -5.46 | -4.81 | 0.14 | Protein |
| 109 | 1ajn | -3.64 | -4.32 | 0.27 | Protein |
| 110 | 1ajp | -3.08 | -3.99 | 0.52 | Protein |
| 111 | 1ajv | -10.59 | -10.94 | 0.21 | Protein |
| 112 | 1ajx | -10.86 | -10.26 | 0.43 | Protein |
| 113 | 1apb | -7.94 | -4.34 | 0.37 | Protein |
| 114 | 1apt | -12.82 | -11.43 | 0.39 | Protein |
| 115 | 1apu | -10.5 | -11.21 | 0.97 | Protein |
| 116 | 1apv | -12.35 | -11.77 | 0.57 | Protein |
| 117 | 1apw | -10.98 | -9.61 | 0.42 | Protein |
| 118 | 1b38 | -9.4 | -10.67 | 0.36 | Protein |
| 119 | 1b39 | -8.97 | -9.87 | 0.33 | Protein |
| 120 | 1b5g | -10.91 | -10.5 | 0.3 | Protein |
| 121 | 1b6j | -10.76 | -12.6 | 0.37 | Protein |
| 122 | 1b6k | -11.88 | -13.02 | 0.31 | Protein |
| 123 | 1b6l | -11.28 | -11.76 | 0.38 | Protein |
| 124 | 1b6m | -11.41 | -11.84 | 0.49 | Protein |
| 125 | 1b9s | -4.39 | -6.25 | 3.11 | Protein |
| 126 | 1b9t | -7.04 | -8.15 | 0.24 | Protein |
| 127 | 1b9v | -5.04 | -9.05 | 0.24 | Protein |
| 128 | 1ba8 | -12.27 | -10.26 | 0.57 | Protein |
| 129 | 1bb0 | -11.4 | -10.2 | 0.47 | Protein |
| 130 | 1bdr | -9.07 | -10.84 | 0.31 | Protein |
| 131 | 1bil | -12.64 | -13.79 | 0.31 | Protein |
| 132 | 1bim | -12.12 | -14.02 | 0.18 | Protein |
| 133 | 1bmm | -9.75 | -10.06 | 0.34 | Protein |
| 134 | 1bmn | -11.58 | -8.68 | 1.59 | Protein |
| 135 | 1bra | -2.5 | -4.92 | 2.88 | Protein |
| 136 | 1bv7 | -12.64 | -14.98 | 0.12 | Protein |
| 137 | 1bv9 | -12.17 | -15.08 | 0.79 | Protein |
| 138 | 1bwa | -10.33 | -14.14 | 0.37 | Protein |
| 139 | 1c29 | -8.74 | -7.37 | 0.22 | Protein |
| 140 | 1c2t | -11.07 | -11.23 | 0.29 | Protein |
| 141 | 1c5c | -9.49 | -7.78 | 0.41 | Protein |
| 142 | 1c83 | -6.59 | -7.46 | 0.22 | Protein |
| 143 | 1c85 | -6.41 | -8.2 | 0.96 | Protein |
| 144 | 1c86 | -7.09 | -8.24 | 0.4 | Protein |
| 145 | 1c87 | -6.59 | -8.52 | 0.19 | Protein |
| 146 | 1c88 | -8.86 | -8.8 | 0.16 | Protein |
| 147 | 1c8k | -6.79 | -7.82 | 0.52 | Protein |
| 148 | 1c9d | -8.71 | -10.19 | 0.12 | Protein |
| 149 | 1cbs | -9.82 | -7.54 | 0.5 | Protein |
| 150 | 1cdg | -3.3 | -2.44 | 0.33 | Protein |
| 151 | 1cf8 | -8.23 | -8.32 | 0.29 | Protein |
| 152 | 1com | -5.44 | -6.22 | 0.45 | Protein |
| 153 | 1cpi | -10.07 | -13.14 | 0.32 | Protein |
| 154 | 1cqp | -6.61 | -6.64 | 0.32 | Protein |
| 155 | 1csc | -9.81 | -12.04 | 0.48 | Protein |
| 156 | 1ctr | -5.84 | -5.46 | 0.33 | Protein |
| 157 | 1ctt | -6.24 | -3.93 | 0.16 | Protein |
| 158 | 1cvu | -10.76 | -6.94 | 0.32 | Protein |
| 159 | 1cw2 | -8.49 | -8.12 | 0.12 | Protein |
| 160 | 1cx2 | -10.94 | -7.16 | 0.24 | Protein |
| 161 | 1cx9 | -9.32 | -8.54 | 0.46 | Protein |
| 162 | 1d3d | -12.55 | -10.78 | 0.41 | Protein |
| 163 | 1d3h | -6.01 | -7.46 | 0.26 | Protein |
| 164 | 1d3t | -7.78 | -9.91 | 1.01 | Protein |
| 165 | 1d4l | -11.92 | -12.48 | 0.32 | Protein |
| 166 | 1d4p | -8.59 | -8.94 | 0.4 | Protein |
| 167 | 1dg5 | -5.53 | -6.75 | 0.24 | Protein |
| 168 | 1dhf | -10.09 | -8.77 | 1.4 | Protein |
| 169 | 1die | -2.92 | -2.78 | 0.16 | Protein |
| 170 | 1dmp | -13.03 | -11.92 | 0.34 | Protein |
| 171 | 1dog | -5.48 | -3.23 | 0.32 | Protein |
| 172 | 1dr1 | -7.59 | -5.29 | 0.32 | Protein |
| 173 | 1drf | -10.15 | -9.2 | 0.23 | Protein |
| 174 | 1dwb | -3.98 | -5.06 | 0.58 | Protein |
| 175 | 1dwc | -10.1 | -8.54 | 0.27 | Protein |
| 176 | 1dwd | -11.14 | -10.4 | 0.48 | Protein |
| 177 | 1dy9 | -10.32 | -10.29 | 0.18 | Protein |
| 178 | 1c85 | -6.41 | -8.2 | 0.96 | Protein |
| 179 | 1eed | -6.54 | -8.49 | 0.82 | Protein |
| 180 | 1ela | -8.66 | -8.54 | 0.43 | Protein |
| 181 | 1elb | -9.75 | -7.19 | 0.32 | Protein |
| 182 | 1eld | -9.13 | -8.63 | 0.19 | Protein |
| 183 | 1ele | -9.33 | -8.76 | 0.85 | Protein |
| 184 | 1ent | -9.55 | -8 | 0.88 | Protein |
| 185 | 1enu | -7.02 | -6.02 | 0.55 | Protein |
| 186 | 1epo | -10.86 | -9.95 | 0.73 | Protein |
| 187 | 1epp | -9.77 | -10.19 | 0.27 | Protein |
| 188 | 1ets | -11.62 | -9.77 | 0.68 | Protein |
| 189 | 1ett | -8.44 | -8.88 | 0.37 | Protein |
| 190 | 1ezq | -12.5 | -11.24 | 1.03 | Protein |
| 191 | 1f0r | -10.58 | -8.8 | 0.55 | Protein |
| 192 | 1f0t | -8.29 | -6.92 | 2.36 | Protein |
| 193 | 1f0u | -9.89 | -8.98 | 1.6 | Protein |
| 194 | 1f3e | -9.25 | -6.52 | 0.1 | Protein |
| 195 | 1fax | -10.07 | -10.6 | 0.52 | Protein |
| 196 | 1fkg | -8.81 | -6.57 | 0.26 | Protein |
| 197 | 1flr | -6.35 | -6.25 | 0.18 | Protein |
| 198 | 1g2k | -10.81 | -12.13 | 0.37 | Protein |
| 199 | 1ghb | -1.71 | -6.13 | 0.26 | Protein |
| 200 | 1gno | -10.57 | -10.1 | 0.29 | Protein |
| 201 | 1gpy | -6.4 | -7.07 | 0.36 | Protein |
| 202 | 1hbv | -8.68 | -10.91 | 0.44 | Protein |
| 203 | 1hdc | -8.17 | -8.03 | 0.82 | Protein |
| 204 | 1hdt | -10.6 | -11.87 | 0.27 | Protein |
| 205 | 1hew | -8.18 | -8.37 | 0.5 | Protein |
| 206 | 1hge | -3.09 | -4.49 | 0.8 | Protein |
| 207 | 1hgh | -3.89 | -4.7 | 0.2 | Protein |
| 208 | 1hgi | -3.65 | -4.83 | 0.89 | Protein |
| 209 | 1hgj | -2.26 | -3.65 | 0.14 | Protein |
| 210 | 1hih | -10.97 | -11.8 | 0.2 | Protein |
| 211 | 1hii | -9.89 | -11.79 | 0.28 | Protein |
| 212 | 1hiv | -12.64 | -13.14 | 0.34 | Protein |
| 213 | 1hos | -11.74 | -13.18 | 0.33 | Protein |
| 214 | 1hpo | -11.82 | -11.14 | 0.39 | Protein |
| 215 | 1hps | -12.66 | -10.94 | 0.32 | Protein |
| 216 | 1hpv | -12.58 | -10.83 | 0.19 | Protein |
| 217 | 1hpx | -12.53 | -13.18 | 0.42 | Protein |
| 218 | 1hri | -5.91 | -7.67 | 0.21 | Protein |
| 219 | 1hrn | -10.94 | -11.82 | 0.22 | Protein |
| 220 | 1hsg | -12.93 | -12.49 | 0.35 | Protein |
| 221 | 1hsh | -11.69 | -12.07 | 0.33 | Protein |
| 222 | 1hte | -7.69 | -8.06 | 0.22 | Protein |
| 223 | 1htf | -11.04 | -9.99 | 0.25 | Protein |
| 224 | 1htg | -13.21 | -12.86 | 0.44 | Protein |
| 225 | 1hvh | -10.81 | -12.18 | 0.45 | Protein |
| 226 | 1hvi | -13.74 | -15.92 | 0.21 | Protein |
| 227 | 1hvj | -14.26 | -15.02 | 0.21 | Protein |
| 228 | 1hvk | -13.8 | -15.48 | 0.52 | Protein |
| 229 | 1hvl | -12.28 | -14.88 | 0.32 | Protein |
| 230 | 1hvr | -12.97 | -12.96 | 0.19 | Protein |
| 231 | 1hxb | -13.48 | -12.76 | 0.29 | Protein |
| 232 | 1hxw | -14.54 | -14.6 | 0.33 | Protein |
| 233 | 1ida | -11.86 | -12.55 | 0.33 | Protein |
| 234 | 1imb | -5.73 | -3.3 | 2.93 | Protein |
| 235 | 1ivb | -4.14 | -5.39 | 1.88 | Protein |
| 236 | 1ivf | -6.66 | -7.62 | 0.88 | Protein |
| 237 | 1k1l | -9.56 | -7.41 | 0.37 | Protein |
| 238 | 1k1n | -8.82 | -9.47 | 0.34 | Protein |
| 239 | 1lyb | -15.57 | -12.36 | 0.22 | Protein |
| 240 | 1mcb | -6.6 | -8.5 | 0.4 | Protein |
| 241 | 1mcf | -7.02 | -8.54 | 0.47 | Protein |
| 242 | 1mch | -7.02 | -8.81 | 0.28 | Protein |
| 243 | 1mcj | -5.16 | -5.92 | 0.38 | Protein |
| 244 | 1mcs | -6.6 | -8.2 | 0.28 | Protein |
| 245 | 1mrk | -6.17 | -5.78 | 0.39 | Protein |
| 246 | 1mtw | -10.07 | -7.84 | 1.01 | Protein |
| 247 | 1nis | -4.08 | -6.66 | 0.19 | Protein |
| 248 | 1nnb | -5.45 | -7.1 | 0.42 | Protein |
| 249 | 1nsc | -4.08 | -7.85 | 0.15 | Protein |
| 250 | 1ola | -9.54 | -10.16 | 0.25 | Protein |
| 251 | 1pdz | -5.03 | -5.8 | 0.38 | Protein |
| 252 | 1pgp | -7.77 | -9.15 | 2.37 | Protein |
| 253 | 1phh | -3.98 | -4.61 | 0.59 | Protein |
| 254 | 1ppc | -8.8 | -8.46 | 0.41 | Protein |
| 255 | 1pph | -8.48 | -6.35 | 1.45 | Protein |
| 256 | 1ppk | -10.44 | -8.91 | 0.44 | Protein |
| 257 | 1ppm | -7.95 | -9.74 | 0.38 | Protein |
| 258 | 1pso | -14.11 | -12.34 | 0.49 | Protein |
| 259 | 1qbr | -14.42 | -14.54 | 0.33 | Protein |
| 260 | 1qbt | -14.49 | -14.61 | 0.45 | Protein |
| 261 | 1qbu | -13.97 | -12.05 | 0.29 | Protein |
| 262 | 1rbp | -9.17 | -7.78 | 0.23 | Protein |
| 263 | 1rgk | -5.87 | -6.82 | 0.22 | Protein |
| 264 | 1rgl | -6.04 | -5.73 | 2.66 | Protein |
| 265 | 1rne | -11.94 | -15.1 | 0.45 | Protein |
| 266 | 1snc | -9.25 | -11.89 | 0.23 | Protein |
| 267 | 1sre | -5.45 | -6.19 | 0.27 | Protein |
| 268 | 1tlc | -10.98 | -10.08 | 0.39 | Protein |
| 269 | 1tng | -4 | -3.93 | 0.5 | Protein |
| 270 | 1tnh | -4.59 | -4.49 | 0.38 | Protein |
| 271 | 1tni | -2.32 | -3.74 | 0.53 | Protein |
| 272 | 1tnj | -2.67 | -3.72 | 0.32 | Protein |
| 273 | 1tnk | -2.03 | -3.81 | 0.36 | Protein |
| 274 | 1tnl | -2.56 | -3.56 | 1.41 | Protein |
| 275 | 1tph | -3.1 | -5.21 | 0.38 | Protein |
| 276 | 1tpp | -7.95 | -6.05 | 0.21 | Protein |
| 277 | 1ulb | -2.8 | -2.84 | 0.15 | Protein |
| 278 | 1uvs | -7.41 | -7.53 | 0.72 | Protein |
| 279 | 2abh | -8.88 | -7.38 | 0.57 | Protein |
| 280 | 2acs | -2.75 | -4.8 | 0.77 | Protein |
| 281 | 2ak3 | -5.25 | -9.83 | 0.44 | Protein |
| 282 | 2cgr | -9.93 | -10.56 | 0.17 | Protein |
| 283 | 2cmd | -6.24 | -7.77 | 0.35 | Protein |
| 284 | 2er7 | -12.27 | -13.64 | 1.03 | Protein |
| 285 | 2ifb | -7.41 | -8.07 | 0.37 | Protein |
| 286 | 2mcp | -7.13 | -6.77 | 0.13 | Protein |
| 287 | 2pk4 | -5.88 | -2.99 | 0.33 | Protein |
| 288 | 2r04 | -8.48 | -7.52 | 0.29 | Protein |
| 289 | 2sim | -8.75 | -6.8 | 0.48 | Protein |
| 290 | 2upj | -10.14 | -10.7 | 0.49 | Protein |
| 291 | 2wea | -8.37 | -6.48 | 0.25 | Protein |
| 292 | 2web | -7.03 | -9.28 | 0.37 | Protein |
| 293 | 2wec | -6.8 | -6.75 | 0.47 | Protein |
| 294 | 2ypi | -6.58 | -7.19 | 0.38 | Protein |
| 295 | 3er3 | -9.68 | -12.89 | 0.88 | Protein |
| 296 | 3er5 | -12.35 | -14.47 | 0.39 | Protein |
| 297 | 3ptb | -6.46 | -5.36 | 0.98 | Protein |
| 298 | 4dfr | -13.23 | -11.4 | 0.3 | Protein |
| 299 | 4er2 | -11.03 | -10.3 | 0.35 | Protein |
| 300 | 4est | -9.55 | -8.08 | 0.51 | Protein |
| 301 | 4hmg | -3.48 | -3.97 | 0.33 | Protein |
| 302 | 4phv | -12.47 | -12.29 | 0.38 | Protein |
| 303 | 4ts1 | -7.64 | -4.81 | 0.2 | Protein |
| 304 | 5cna | -2.73 | -3.71 | 0.77 | Protein |
| 305 | 5enl | -5.24 | -6.59 | 1.05 | Protein |
| 306 | 5hvp | -10.51 | -12.9 | 0.22 | Protein |
| 307 | 5tim | -3.14 | -3.09 | 0.54 | Protein |
| 308 | 6enl | -4.14 | -5.53 | 0.51 | Protein |
| 309 | 6rnt | -8.67 | -7.26 | 0.31 | Protein |
| 310 | 6tim | -4.37 | -7.59 | 0.32 | Protein |
| 311 | 7dfr | -10.09 | -10.73 | 0.29 | Protein |
| 312 | 7gpb | -7.53 | -7.64 | 0.94 | Protein |
| 313 | 7tim | -7.35 | -7.01 | 0.12 | Protein |
| 314 | 8gpb | -4.91 | -5.96 | 0.53 | Protein |
| 315 | 9hvp | -11.38 | -13.9 | 0.51 | Protein |
| 316 | 2ikj | -8.86 | -10.08 | 0.18 | Protein |
| 317 | 2ikh | -7.48 | -4.72 | 0.19 | Protein |
| 318 | 2ikg | -8.46 | -9.12 | 0.19 | Protein |
| 319 | 1e2j | -7.2 | -6.17 | 2.68 | Protein |
| 320 | 1k1i | -8.69 | -7.86 | 0.21 | Protein |
| 321 | 1hxd | -9.9 | -8.71 | 0.64 | Protein |
| 322 | 1k1j | -10.41 | -8.66 | 0.2 | Protein |
| 323 | 1k1l | -9.74 | -7.81 | 0.21 | Protein |
| 324 | 1afk | -9 | -13.4 | 0.32 | Protein |
| 325 | 1afl | -8.53 | -9.44 | 0.33 | Protein |
| 326 | 1anf | -7.442 | -5.87 | 0.28 | Protein |
| 327 | 1c84 | -6.79 | -9.06 | 0.13 | Protein |
| 328 | 1d3p | -10.07 | -11.08 | 0.25 | Protein |
| 329 | 1eap | -8.47 | -8.64 | 0.16 | Protein |
| 330 | 1elc | -9.76 | -9.51 | 0.26 | Protein |
| 331 | 1etr | -10.11 | -9.43 | 0.20 | Protein |
| 332 | 1nsd | -7.23 | -7.8 | 0.19 | Protein |
| 333 | 1tmt | -8.51 | -7.93 | 0.15 | Protein |
| 334 | 2msb | -3.94 | -2.21 | 1.58 | Protein |
| 335 | 3cla | -6.037 | -3.88 | 0.22 | Protein |

^a^ Protein Data Bank ID of the drug target

^b^ Experimental binding free energy (in kcal/mol) of the native target-ligand complex.

^c^ Predicted binding free energy (in kcal/mol) of the docked target-ligand complex.

^d^ Root Mean Square Deviation (in Å) between the crystal structure and the docked structure.
